# Hostile work climate: gender barriers in the European energy R&I workforce

**DOI:** 10.3389/fsoc.2026.1800176

**Published:** 2026-06-03

**Authors:** Maria Camilla Fraudatario, Lucio Pisacane, Marco Cellini, Cloe Mirenda

**Affiliations:** Institute for Research on Population and Social Policies, National Research Council (IRPPS-CNR), Rome, Italy

**Keywords:** gender inequalities, renewable energy sector, research and innovation workforce, workplace dynamics, cluster analysis

## Abstract

Achieving climate neutrality by 2050 demands a fundamental transformation of the energy sector, which currently accounts for 76.6% of global greenhouse gas emissions. Meeting these targets requires attention not only to the green technologies produced by research and innovation (R&I), but also to who drives these innovations and under what working conditions. Despite progress made over recent decades, women remain systematically underrepresented in the energy sector, owing to deeply rooted structural dynamics: male-dominated organizational cultures, recruitment and evaluation processes shaped by gender bias, and professional climates that hinder women's full participation and career advancement. This study examines the R&I workforce in the energy sector, a domain in which gender imbalances have thus far received limited scholarly attention. A cross-national survey was conducted among employees of European public and private universities and research organizations, structured around sociographic and professional variables, organizational context variables, and four validated psychometric scales—Work-Related Quality of Life, Perceived Subtle Gender Bias Index, Just Energy Transition Knowledge Production, and Workplace Diversity Climate—comprising a total of 43 items. The data enabled examination of: i) perceptions regarding institutional support for women's career trajectories and the handling of both overt inequalities and implicit biases; ii) everyday workplace practices, such as professional recognition, empowerment, and well-being, as key determinants in fostering a genuine commitment to sustainability. Methodologically, the latent structure of the 43 items was identified through exploratory and confirmatory factor analysis, yielding nine composite factors subsequently used to perform a cluster analysis. The results identify two contrasting profiles: a Critical Cluster, predominantly female, in which the working environment is perceived as hostile and gender discrimination manifests in multiple forms, including implicit bias, verbal harassment, and inadequate support for work-life balance; and a Neutral Cluster, predominantly male and concentrated in the renewable energy, characterized by a more positive assessment of the organizational climate and limited awareness of gender stereotypes and discriminatory practices. This polarization calls into question the inclusivity and equity of the current R&I system in the energy sector. The study provides an empirical contribution to the debate on inclusive energy transitions and puts forward policy recommendations to strengthen gender equity measures within R&I organizations.

## 1 Introduction

Research and Innovation (R&I) play a fundamental role in developing and implementing climate mitigation strategies (Blanco et al., 2022; Aleluia Reis et al., 2023). The European Commission has long recognized the strategic importance of R&I for generating sustainable solutions to the multiple social, economic, and environmental challenges of our time (European Commission, Directorate-General for Research and Innovation, Empirica, Portia, Fraunhofer-IAO CeRRI, DIW-Econ, ÖGUT, GDCC, 2024).

The goal of climate neutrality by 2050 puts a spotlight on the energy sector, as it currently accounts for 76.6% of global greenhouse gas emissions (Clarke et al., 2022; Climate Watch Historical GHG Emissions, 2026).

Achieving climate targets will therefore depend on the rapid deployment of existing green technologies—improving their efficiency and affordability (Aleluia Reis et al., 2023; Cervantes et al., 2023)—and on advancement of emerging solutions, including hydrogen and next-generation energy storage solutions.

Yet meeting these objectives requires attention not only to what R&I produces, but also to who drives it and under what conditions. Despite progress made over recent decades, women remain systematically underrepresented in the energy sector (Feenstra and Kottari, 2024; Striebing et al., 2024) and in the R&I organizations operating within it. This imbalance reflects structural dynamics embedded in workplace contexts: male-dominated organizational cultures, recruitment and evaluation practices shaped by gender bias, and professional climates that hinder women's full participation and career advancement (Kinahan et al., 2020).

Tackling gender inequality leads to fairer participation, a broader pool of talents dedicated to the green transition (Pearl-Martinez and Stephens, 2016; Hoicka, 2023; Feenstra and Kottari, 2024), and ultimate to better science and innovation outputs. Gender balance in scientific careers further supports mitigation goals, as women often show greater environmental awareness and are more likely to translate such sensitivity into concrete actions (Allison et al., 2019). Indeed, several studies indicated that women's participation in research teams enhances research performance, producing more innovative publications (Yang et al., 2022), higher-quality patents (Hofstra et al., 2020; Zhang and Zong, 2023), and more inclusive work environments (Lazoroska et al., 2024). Despite the key role that broadening participation in R&I for the energy transition plays in relation to sustainability, only a limited number of studies have explored gender imbalance in this sector (Smith et al., 2019; C. F. F., Cities Finance Facility, 2021; European Commission, Directorate-General for Research and Innovation, Empirica, Portia, Fraunhofer-IAO CeRRI, DIW-Econ, ÖGUT, GDCC, 2024), and those available provide limited explanatory insight. Although extensive research addresses women's underrepresentation in STEM, little is known about the specific barriers faced by professionals in renewable energy R&I. Existing literature considers women in boards and management of large energy companies (Carlsson-Kanyama et al., 2010), decision-making in renewables and energy policymaking (IRENA, 2023; IEA, 2024).

One of the reasons for this limited understanding of internal organizational dynamics is certainly linked to the difficulty of conducting research and analysis in professional environments and organizations that are often reluctant to share information about workplace climate, organizational practices, and career development and progression processes. As a result, existing studies tend to be either strictly qualitative in nature, offering highly specific in-depth analyses of single organizations (van den Brink and Benschop, 2014; O'Connor, 2020), or, conversely, very large-scale investigations with a low level of detail and limited capacity to explain gender mechanisms within research organizations (European Commission, Directorate-General for Research and Innovation, Empirica, Portia, Fraunhofer-IAO CeRRI, DIW-Econ, ÖGUT, GDCC, 2024; EIGE, 2025). In addition, capturing the dynamics that hinder women's entry, retention, and career progression in STEM (Wentling and Thomas, 2009) requires indicators and data that are often unavailable or not systematically collected challenges that stem from inherent difficulties in gender monitoring. Such monitoring frequently relies on quantitative sex-disaggregated data. However, understanding gender dynamics also requires qualitative indicators, such as perceptions and experiences, which are more difficult to operationalize and are typically gathered through surveys or focus groups (Sánchez-López et al., 2024).

This paper addresses this gap by presenting an exploratory cross-national survey on gender barriers in energy R&I organizations. The survey was designed in the context of the EU funded gEneSys project, to gauge employees' job satisfaction and assess the organizational culture and workplace dynamics. In particular, the aim of the survey was to understand to what extent gender barriers and stereotypes still permeate the R&I sector in the context of the energy transition and assess the presence or absence of an inclusive workplace climate within public and private R&I organization engaged in energy transition related research. Results show how organizational practices and workplace cultures reproduce gendered exclusions, and we discuss the implications for policy and organizational change in the context of the energy transition. The survey builds on a recent study commissioned by CINEA, European Climate, Infrastructure and Environment Executive Agency, to assess gender balance in the R&I field in the energy sector (European Commission, Directorate-General for Research and Innovation, Empirica, Portia, Fraunhofer-IAO CeRRI, DIW-Econ, ÖGUT, GDCC, 2024). It was distributed to professionals engaged in R&I activities across energy-related fields within public and private organizations including research centers, academic departments, national/local agencies, companies, and businesses.

The paper is structured as follows: Section 2 reviews the available literature and situates our contribution within it. Section 3 outlines the questionnaire's design, administration, and the methodology used for data analysis. Section 4 presents the results. Section 5 concludes with a discussion of the findings, their implications and limitations along with key policy recommendations.

## 2 Theoretical framework

Despite efforts to reduce gender inequalities in R&I, women researchers continue to face structural barriers and workplace dynamics that hinder their full participation, especially in STEM fields. The scientific literature has addressed this dynamic through quantitative lenses, operationalizing gender imbalance via sex-disaggregated data and differential participation rates across research sectors and scientific disciplines (Ceci and Williams, 2011). The *She Figures 2024* report confirms that women remain underrepresented in STEM fields, where they are more likely to hold part-time contracts, earn low salaries, and face barriers to career advancement (European Commission, Directorate-General for Research and Innovation, 2025).

These inequalities are particularly salient within the energy sector. Contrary to expectations that the renewable energy transition would provide a strategic opportunity to narrow gender gaps in techno-scientific domains (Webb et al., 2021; IRENA, 2023), gender gaps persist even within emerging roles in the sector (Striebing et al., 2024; European Commission, Directorate-General for Research and Innovation, Empirica, Portia, Fraunhofer-IAO CeRRI, DIW-Econ, ÖGUT, GDCC, 2024). Indeed, women constitute a mere 22% of the energy R&I workforce (European Commission, Directorate-General for Research and Innovation, Empirica, Portia, Fraunhofer-IAO CeRRI, DIW-Econ, ÖGUT, GDCC, 2024). Given that the success of the energy transition hinges upon the availability of highly skilled and committed R&I professionals (Soriano and Mulatero, 2011), such imbalances carry profound strategic implications, particularly regarding social equity.

From a regulatory perspective, the European Commission has institutionalized gender equality as a formal requirement under Horizon Europe through mandatory Gender Equality Plans (GEPs). Simultaneously, the United Nations Sustainable Development Goals recognize gender equality (SDG 5), access to clean energy (SDG 7) and climate action (SDG 13) as structurally interdependent objectives. Notwithstanding these institutional commitments, empirical evidence suggests that significant barriers persist, especially within energy-related R&I organizations. These barriers translate into a reduced participation, for instance, in the engineering and technology field, which includes most researchers in the energy sector, women hold 17.9% of full professor positions (European Commission, Directorate-General for Research and Innovation, Empirica, Portia, Fraunhofer-IAO CeRRI, DIW-Econ, ÖGUT, GDCC, 2024).

To move beyond a predominantly numerical perspective on gender imbalance in R&I, a growing body of scholarship has adopted a more context-sensitive approach that examines the interaction between organizational structures, management practices, and workplace culture. Within this framework, organizations are conceptualized as social systems in which formal rules, informal norms, and institutional cultures jointly shape access to positions, career progression, and the everyday experiences of personnel (Acker, 1990; Lamont, 2009). This literature highlights how structural factors—such as hierarchical arrangements, contractual typlogies, and evaluation procedures—and organizational climate interact with relational and cultural dynamics, thereby shaping the opportunities and constraints encountered by women within research environments (Fox, 2001; Lamont, 2009; Bird, 2011).

While quantitative indicators are effective in identifying patterns of representation, they offer limited insight into the mechanisms through which gender inequalities are produced and reproduced. Qualitative indicators, such as perceptions of fairness, transparency in recruitment and promotion processes, access to informal networks, experiences of bias or exclusion, and the availability and use of work-life balance measures, are therefore essential for capturing how organizational practices and cultures influence gender dynamics. Integrating quantitative and qualitative indicators is crucial not only for assessing women's presence in energy research, but also for understanding the systemic and relational processes that shape their career trajectories and professional experiences (Nielsen, 2016).

Women's persistent underrepresentation in R&I arises from a complex interplay of structural, cultural, and economic factors. Studies identify universal barriers to women's advancement, especially during transitions to stable positions rooted in competitive, individualized progression systems (Kinahan et al., 2020; Ghaempanah and Khapova, 2023). Gender inequalities in research careers emerge from several factors, including gender biased evaluations, limited mentoring, workplace discrimination, and disparities in promotion and pay, exacerbated by caregiving challenges (Kinahan et al., 2020; Harris et al., 2024). The “leaky pipeline” (women's gradual exit from scientific careers) worsens after childbirth, as family obligations clash with high productivity demands (Weisgram and Diekman, 2017; Sebastián-González et al., 2023).

Moreover, the interaction between situated socio-cultural practices and gender (Saifuddin et al., 2019) reinforces bias and further limit women's career opportunities in research. Notably, Prozesky and Beaudry (2019) question the conventional assumption that caregiving responsibilities are the primary barrier to African women researchers' mobility. Instead, they highlight the influence of gender-based restrictions, including patriarchal norms that limit women's social interactions and freedom of movement. Similarly, Krilić et al. (2018) argue that the difficulty of reconciling care with academic careers reflects institutional structures that marginalize reproductive labor. The academic culture, built upon the male ideal worker model, compels women researchers to strategically defer motherhood and navigate a double burden of labor. These dynamics persist across diverse European national contexts, regardless of the relative generosity of formal work-life balance policies.

Gender disparities are also prominent in Northern European countries, with segregation affecting fields, networks, and work environments (Griffin, 2022). Analyzing and addressing these inequalities requires context-specific solutions at both national (e.g., research investment) and organizational (e.g., departmental equity policies) levels (Griffin, 2022).

These issues intensify in energy-system research, historically STEM-dominated, where women's representation is notably lower than average (Smith et al., 2019). At the European level, women account for only 8–10% of inventors in energy-related patent classes, compared to 16% across all technological fields (IEA, 2020). Even in the solar industry, where women's participation (40%) exceeds that in wind energy (21%) (IRENA, 2022), inequalities remain. Most women are primarily employed in administrative roles (58%), while only 32% hold STEM-related positions (IRENA, 2022). Recent studies show that the energy sector, both fossil and renewables, faces major socio-economic inequalities (Baruah and Gaudet, 2022; Lazoroska et al., 2024), with a predominantly male workforce (Cellini et al., 2025). This is due to two main factors: the gender imbalance in STEM pipelines, and work environments that fail to support female participation (European Commission, Directorate-General for Research and Innovation, Empirica, Portia, Fraunhofer-IAO CeRRI, DIW-Econ, ÖGUT, GDCC, 2024; Striebing et al., 2024). Regarding the former, extensive scholarship has documented how technology is historically constructed as a masculine domain (Faulkner, 2001; Wajcman, 2006), reproducing sociotechnical imaginaries that discourage women from identifying with techno-scientific careers (Haraway, 2013). These dynamics become embedded in institutional evaluation and selection practices, aligning criteria of excellence with implicitly male profiles (Lamont, 2009).

The latter factor concerns the impact of hostile workplace climates, which further contribute to higher attrition rates among women. Studies indicate that women often leave due to discrimination or harassment, while men tend to exit for better opportunities (Spoon et al., 2023; Litzellachner et al., 2024). Inequities extend beyond structural barriers to power dynamics: few women occupy senior or leadership roles, limiting their decision-making influence, and their participation as principal investigators or co-investigators remains significantly below that of men (Sánchez-López et al., 2024).

Despite a marginal increase in the last few years, the share of women seeking energy-related research grants remains low, and funds allocated to them generally lag those for men. Barriers include the size and timing of funding calls and the broader academic culture and evaluation criteria. Additional challenges involve minimal support for those with caregiving responsibilities or fragmented career paths, as well as difficulties forming industry partnerships in a male-dominated sector (Smith et al., 2019).

As a result, gender blindness in energy R&I has a dual impact: it wastes talent by confining women to subordinate roles with slow career progression or pushing them to leave the field, and it weakens research quality. Gender perspectives are often dismissed as irrelevant, as illustrated by studies on photovoltaic cell placement, in which the social dimensions and end-use patterns of technologies are systematically overlooked (Sánchez-López et al., 2024).

However, a growing body of research challenges this assumption, emphasizing that the ecological transition cannot be regarded as a gender-neutral process. Green technologies, far from being merely technical artifacts, embody the values, priorities and perspectives of those who design and implement them (Geels, 2004; Clancy, 2016; Jenkins et al., 2018; Bell et al., 2020). In a historically male-dominated, technocentric energy sector, technology solutions may overlook how women and other socially marginalized groups access, use and consume energy, potentially exacerbating inequalities in decarbonisation pathways (Sovacool et al., 2022). The lack of targeted interventions in organizational cultures and decision-making processes within many R&I institutions puts the ecological transition at risk of reproducing the same power hierarchies that characterized the fossil fuel energy system, thus limiting the transformative potential of emerging technologies (Bell et al., 2020; Pearl-Martinez and Stephens, 2016).

Against this backdrop, the scientific literature converges on the view that ecological transition requires inclusive approaches that systematically integrate gender and minority dimensions into energy research programmes (Cannon and Chu, 2021; Ryan, 2014; Sovacool, 2014), while drawing on a broad talent pool grounded in gender inclusion and other forms of diversity (Pearl-Martinez and Stephens, 2016).

In this regard, Hoicka (2023) argues for the need to strengthen energy research programs through a multi-level approach. First, integrating equity principles into all stages of the research process is critical not only to ensure more equitable access to scientific and professional opportunities, but also to incorporate the social dimensions of the most vulnerable populations into STEM agendas. Second, creating inclusive environments, through targeted recruitment strategies that engage researchers from different backgrounds and the adoption of mentoring practices that support the experiences of historically underrepresented groups, fosters greater equity in the field (Hoicka, 2023).

This transformation requires a deep cultural shift in research institutions, which are still marked by structural barriers and discrimination. Such barriers manifest in unequal access to publication and funding, as well as in recruitment, retention, and promotion processes, disproportionately affecting women and other underrepresented groups (Smith et al., 2019; Wilgosh et al., 2022).

Prevailing cultural norms also shape the work environment, creating mixed experiences for women in energy research (Smith et al., 2019). While Gender Equality Plans and diversity commitments mark progress, actual implementation often misaligned with the best practices they theoretically promote (Lazoroska et al., 2024; Picardi et al., 2023). Unwelcoming conditions persist where long hours are valued, contracts are short-term, and caregiving needs go unmet. Consequently, “invisible women” remain a reality in energy research (Smith et al., 2019).

## 3 Materials and methods

This study examines the workplace climate within European public and private universities and research organizations operating in the energy sector, with the aim of exploring organizational cultures and the workplace dynamics that shape professional experience.

A growing body of literature frames the energy transition as an inherently social challenge, requiring, among other things, the reconfiguration of work environments through an equitable and inclusive lens (Feenstra and Kottari, 2024; Picardi, 2022; Hoicka, 2023).

Building on this theoretical premise, the study draws on the opinions and perceptions of respondents to investigate two key aspects. The first concerns gender equity: specifically, the extent to which universities and research organizations support women's career trajectories and address both overt inequalities and implicit biases. This issue is salient given the documented need for skilled personnel and greater diversity in the workforce engaged in the renewable energy sector (Feenstra and Kottari, 2024).

The second aspect relates to practices focused on professional recognition, empowerment and wellbeing, which are considered decisive drivers in fostering a concrete commitment to sustainability.

The analytical approach is exploratory and descriptive in nature: the results provide an empirical mapping of the current state of universities and research organizations, highlighting the structural and cultural factors that respondents perceive as enablers or barriers in their professional contexts.

### 3.1 Research design and tool development

This study employs a quantitative cross-sectional research design. Data was collected via a survey targeting the R&I workforce within the energy sector. The survey instrument was developed through an iterative process: following the initial formulation by the National Research Council (CNR) research team, the tool underwent a formal review by the gEneSys consortium partners. Subsequently, a pilot study was conducted with a purposive sample of *N* = 11 individuals. Participants were selected to ensure heterogeneity in terms of gender, nationality, and institutional background. The pilot phase led to the optimization of the administration protocol and the refinement of specific survey items.

### 3.2 Sampling strategy and data collection

The target population consists of professionals active in R&I within the European energy sector. Due to the absence of a comprehensive registry of energy researchers, a mixed sampling strategy was adopted. First, a systematic stakeholder mapping identified key public and private institutions. Second, this was supplemented by non-probability snowball sampling, leveraged the gEneSys consortium's established networks in Italy, Germany, Poland, and the United Kingdom. This approach facilitated access to professional networks that are otherwise difficult to reach, thereby enhancing the sample's representativeness. The survey was administered via the LimeSurvey platform between September and November 2024.

### 3.3 Survey structure

The questionnaire is structured into three main analytical sections designed to explore the intersection of ascribed variables and organizational dynamics:

- *Sociographic and Professional Variables* (7 items), to profile the sample according to gender, age, employer type, contractual status, caregiving responsibilities, career stage (seniority), and educational level.
- *Organizational Context* (3 items) operationalized through the specific energy and technology sector, country, and organizational size.
- *Attitudinal Psychometric Scales* (43 items), using validated Likert scales to measure the following latent constructs:
  ° Work-Related Quality of Life (WRQoL), adapted from Easton and Van Laar (2018), this 12-item scale assesses dimensions such as empowerment, recognition, and perceived workplace flexibility.
  ° *Perceived Subtle Gender Bias Index* (PSGBI), developed by Tran et al. (2019), this 21-item scale measures individual awareness of subtle gender-based discrimination.
  ° *Perspective for Just Energy Transition Knowledge Production Scale* (JETKP), an original 6-item scale developed for this study to assess attitudes toward policies aimed at fostering inclusivity within the energy sector.
  ° *Workplace Diversity Climate Scale* (WDC), adapted from Ward et al. (2022), this 4-item scale measures the perceived organizational openness toward diverse professional backgrounds.

The overall architecture of the instrument was designed to capture researchers' perceptions of the occupational and organizational dynamics in which they are embedded.

### 3.4 Methods of analysis

The dataset (Cellini et al., 2026) provides an empirical foundation for investigating the R&I workforce within the European energy sector, and individual perceptions of the respective organizational climate. The analytical protocol was structured into three sequential phases: dataset preparation and exploratory descriptive analysis; multidimensional analysis of psychometric scales; profiling of the workforce through cluster analysis techniques.

In the initial phase, the dataset underwent rigorous verification and cleaning procedures. Within the original sample of *N* = 358 respondents, missing data were identified in 9.8% of the observations. These cases were removed via listwise deletion, resulting in a final analytical sample of *n* = 323 units. This methodological decision was preferred over imputation techniques, such as mean or maximum likelihood imputation, to prevent the introduction of systematic bias into the estimates (Enders, 2022). Following this verification, descriptive analyses were conducted to explore the sociodemographic and organizations characteristics of the sample (see the Data Overview section).

The second phase involved exploring the latent structure of the scale item batteries to identify key dimensions, which served as a preparatory for the third phase involving cluster analysis. For this purpose, Exploratory Factor Analysis (henceforth, EFA) (Fabrigar and Wegener, 2012) was employed to determine the number of latent constructs (Suhr, 2006), followed by Confirmatory Factor Analysis (henceforth, CFA) (Harrington, 2009) to validate the emerging models. Specifically, items of the WRQoL, WDC, and JETKP scales were subjected to a sequential EFA-CFA procedure. This approach was necessary since the first two scales used only a selection of items from existing instruments, while the JETKP scale was developed *ex novo* for this study. Conversely, the PSGBI scale items were tested exclusively via CFA, following the indications of Tran et al. (2019) and utilizing the full set of questions in accordance with the authors' guidelines. The dimensionality reduction process allowed the original information to be synthesized into nine latent factors. Table 1 lists the factors extracted for each set of batteries, specifying the reference items and the identified constructs.

**Table 1:** List of the extracted factors.

Extracted factor	Construct's items	Construct
*WRQoL_F1*	influence; abilities; goals	*Work self-efficacy*
*WRQoL_F2*	acknowledgment; skill development; decision involvement; career opportunity; needs met; safe environment	*Support and recognition*
*WRQoL_F3*	working hours; flexibility	*Organizational flexibility*
*PSGBI_F1*	biases; verbal_prevarication; respect; ambitiousness; subordination; treat_women; support_male; meeting; bias_aknowledgement	*Gender bias*
*PSGBI_F2*	feedback; collegial; relationship; ideas; support_people; feel_valued	*Workplace cohesion*
*PSGBI_F3*	mentoring_informal; mentoring_formal; mentoring_senior	*Mentorship and professional guidance*
*PSGBI_F4*	attuned; support_balance; policy_equity	*Equity-driven climate*
*JETKP_F1*	diversity; culture; favoring_groups; society_representation; male_domination; minorities	*Policy advocacy for inclusivity in the energy sector*
*WDC_F1*	managing_backgrounds; accepted_backgrounds; hiring_practices; retain_diversity	*Diversity and inclusion practices*

Validity tests for the EFA procedure (see Annex 1—Methodology full description) confirmed the adequacy of the sample and the robustness of inter-variable correlations, as supported by the Kaiser-Meyer-Olkin (KMO) measure and Bartlett's test of sphericity. The determination of the number of factors was corroborated by eigenvalue analysis and Parallel Analysis. Key statistical fit indices, including the Standardized Root Mean Square Residual (SRMR), the Root Mean Square Error of Approximation (RMSEA), and the Tucker-Lewis Index (TLI), were found to be fully consistent with methodological acceptability thresholds.

Regarding the CFA, the values for the Comparative Fit Index (CFI), TLI, RMSEA, and SRMR collectively indicated a good fit between the data and the theoretical model. Although a slight deviation was observed in the JETKP model (RMSEA = 0.1), the RMSEA confidence intervals for all other dimensions remained consistently below the critical cut-off of 0.08, confirming an acceptable fit. Overall, the evidence emerging from the CFA strongly validates the proposed factor structures, with fit indices meeting standard parameters.

In the final phase, a Cluster Analysis (CA) was performed on the nine factor scores to identify latent profiles within the R&I workforce of the energy sector. The analytical objective of CA is to maximize within-cluster homogeneity while simultaneously maximizing between-group heterogeneity (Kaufman and Rousseeuw, 2009).

The procedure was structured into four sequential steps. First, the factor scores derived from the CFA were standardized (z-scores). This pre-processing step was essential to normalize the measurement scales across all dimensions, ensuring that each factor contributed with equal metric weight to the distance computation.

Subsequently, the optimal number of clusters (*K*) was determined through the application of the Elbow Method and the Gap Statistic. Both criteria provided consistent evidence, suggesting a two-cluster solution as the most parsimonious compromise between model complexity and descriptive capacity.

The third step involved the execution of the non-hierarchical *K*-means algorithm. Using Euclidean distance as the proximity metric, the algorithm iteratively grouped observations by minimizing within-cluster variance, assigning respondents (Cluster 1 = 131; Cluster 2 = 192) based on the similarity of their profiles across the latent constructs.

Finally, the resulting solution underwent a rigorous validation process. Although the Average Silhouette Width (0.28) reflects a partial overlap in the border regions between groups, the discriminant validity of the partition was confirmed by analyses of variance (ANOVA) (see Annex 1—Methodology full description). The tests demonstrated statistically significant differences (*p* < 0.05) between the centroids of the two clusters for eight of the nine factors examined. The exception was JETKP_F1, which, with a marginal statistical significance (*p* = 0.026), exhibited the lowest segregating power (*F* = 5.0; η^2^ = 0.02). This suggests that while the clusters differ profoundly regarding organizational wellbeing and perceptions of gender bias, they exhibit a more homogeneous distribution in terms of perspectives on inclusivity policies in the energy sector. To complete the characterization of the profiles, percentage distributions for key socio-demographic variables were computed, which are discussed in the following section.

## 4 Results

### 4.1 Data overview

This section outlines the profile of the R&I workforce, providing an analytical focus on gender-based differences. The composition of the sample indicates a female participation rate of 42% compared to a male component of 58%. This distribution appears consistent with the data reported in the She Figures (European Commission, Directorate-General for Research and Innovation, 2025). The geographical distribution of the sample indicates (Table 2) that most researchers of both genders are concentrated in Southern Europe (women 55.9%; men 57.8%). Notably, a slight overrepresentation of female researchers is observed in Eastern Europe (women 10.3% vs. men 6.4%) and Western Europe (women 18.4% vs. men 15%), whereas male researchers show a higher prevalence in Northern Europe (men 20.9% vs. women 15.4%).

**Table 2:** Region (%).

Region	Women	Men
Eastern Europe	10.3	6.4
Northern Europe	15.4	20.9
Southern Europe	55.9	57.8
Western Europe	18.4	15.0

The analysis of age cohorts (Table 3) highlights divergent trends between genders. Women are predominantly concentrated in mature age groups, with the 41–45, 46–50, 51–55, and 56–60 classes collectively accounting for 63.8% of respondents. Conversely, the male distribution appears more polarized: 32.1% of respondents are situated within the intermediate age classes (36–45 years), while 29.9% fall into the senior classes (51–60 years). Younger generation of researcher (26–35 years) are the least represented in the sample, constituting 16.9% of the female workforce and 13.3% of the male workforce, respectively.

**Table 3:** Age class (%).

Age class	Women	Men
>70	0.7	1.6
66–70	4.4	2.3
61–65	12.5	10.2
56–60	16.9	14.4
51–55	16.1	15.5
46–50	16.1	10.2
41–45	14.7	16.6
36–40	11.8	15.5
31–35	10.3	6.9
26–30	6.6	6.4
22–25	4.4	0

In terms of educational background, the sample is homogeneous, as approximately 70% of respondents hold a doctoral degree (ISCED 8). However, this educational uniformity does not translate into identical career progression across genders. The demographic configuration reflects significant differences in seniority levels (Table 4), with 79.1% of men holding senior positions (over 7 years of experience) compared to 67.6% of women. Furthermore, women are more prevalent in junior positions (up to 4 years of experience), where they stand at 18.3% against 10.7% for their male counterparts.

**Table 4:** Seniority (%).

Seniority	Women	Men
Senior (7+ years)	67.6	79.1
Middle career (5–7 years)	13.9	10.1
Junior (0–4 years)	18.3	10.7

Regarding research profile (Table 5), approximately 70% of the workforce for both genders is classified as Researcher or Technologist, and about 20% hold roles as Team Manager or Supervisor. Nevertheless, disparities emerge in top-level positions (Director or Board Member), which are occupied by 6.4% of men compared to 4.4% of women. Additionally, women are overrepresented in the role of Research Assistant (5.9% vs. 1.6%) and are almost entirely absent among technicians (0.7%).

**Table 5:** Research profile (%).

Research profile	Women	Men
Researcher or technologist	69.1	68.9
Team manager/supervisor	16.1	17.1
Director/board member	4.4	6.4
Technician	0.7	3.2
Research assistant	5.9	1.6
Other	3.6	2.6

The contractual agreements reveal a prevalence of permanent contracts, held by 81% of men and 76% of women. Consequently, women are more exposed to contractual precariousness, with 22.9% of them hired on fixed-term or apprenticeship contracts compared to 17% of men. Lastly, almost the entire population (90% of respondents) work in universities or research organizations, both public and private.

### 4.2 Energy sector and renewable technologies

**Figure 1:**
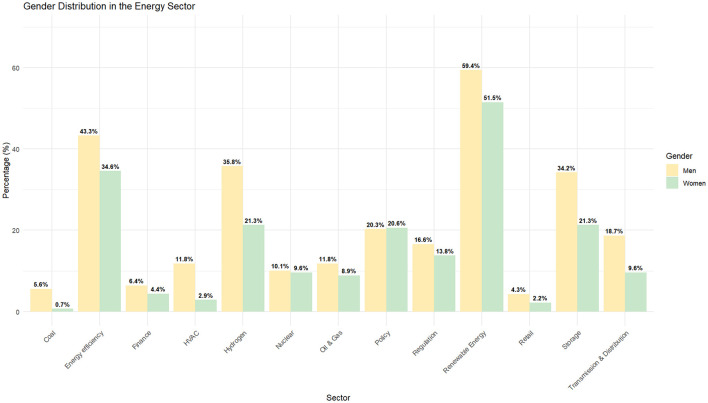
Gender distribution in the energy sector (%).

The survey examined the energy sectors and related renewable technologies as areas of specialist expertise among respondents. Analysis of the workforce concentration across various segments (Figure 1) yields two key insights. First, research agendas and programs are predominantly focused on renewable energy, energy efficiency, storage, hydrogen, policy and regulation, and transmission and distribution. A residual proportion is employed in the retail sector or fossil fuels (oil & gas, and coal).

Second, the findings confirm that women represent a smaller proportion than men across nearly all sectors among respondents; this includes the renewable energy, despite its reputation as the most attractive field for female professionals. In this context, men account for approximately 60%, compared to 52% of women. A marginal difference is observed in the energy policy sector, which involves approximately 21% of women and 20% of men.

**Figure 2:**
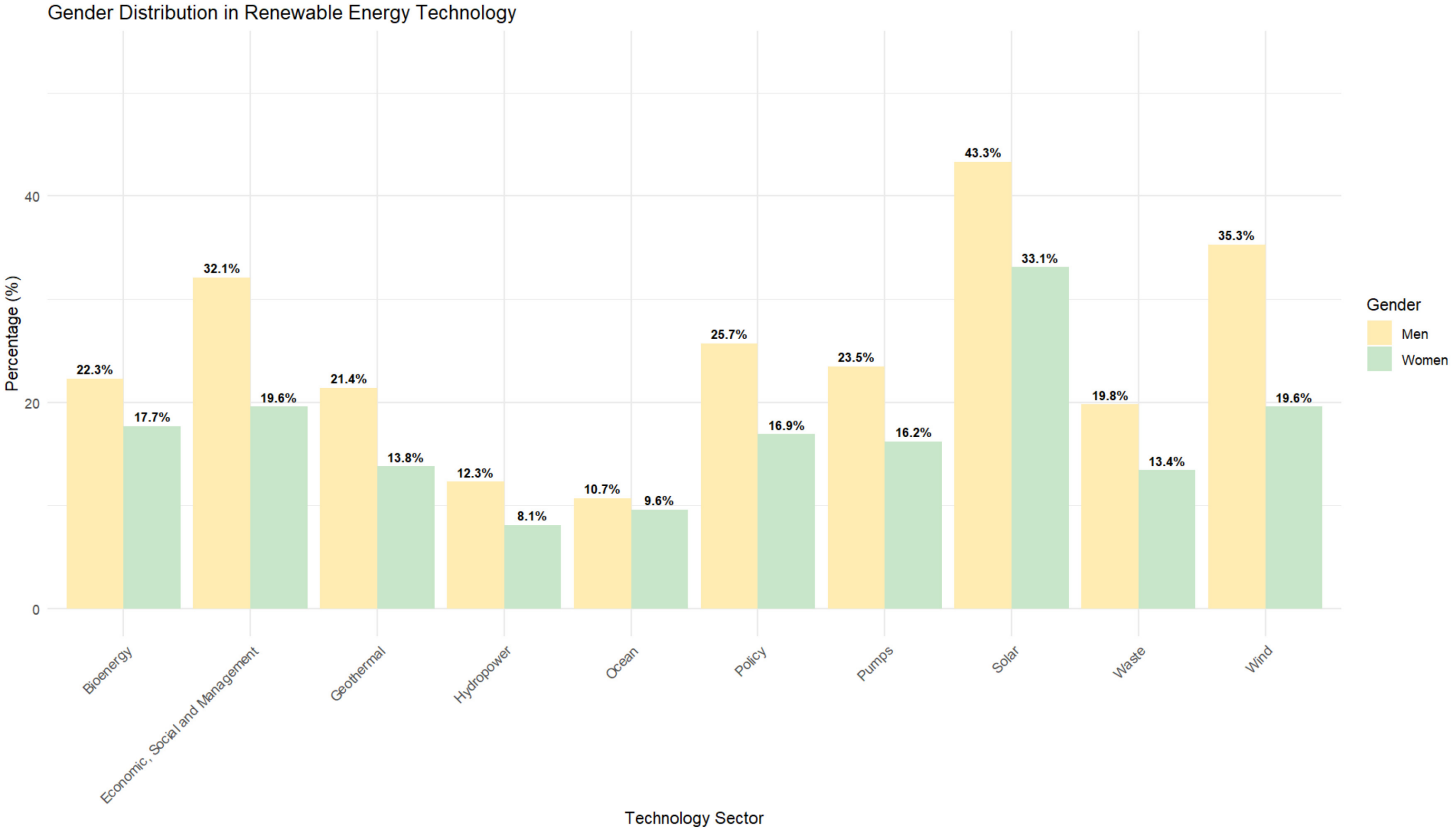
Gender distribution in renewable energy technology (%).

The renewable energy is experiencing significant growth within research organizations; therefore, the specific technologies underlying development and research activities have been further analyzed (Figure 2). Most efforts are aimed at solar and wind technologies, accompanied by advances in economic, social, and management studies, as well as policy.

Isolating the distribution of women, consistent with current scientific evidence (Baruah and Gaudet, 2022; IRENA, 2019a), higher involvement is observed in the solar (33%), followed by the economic, social and management (19.6%) and bioenergy (17.7%).

### 4.3 Cluster analysis: patterns of organizational climate perceptions

**Figure 3:**
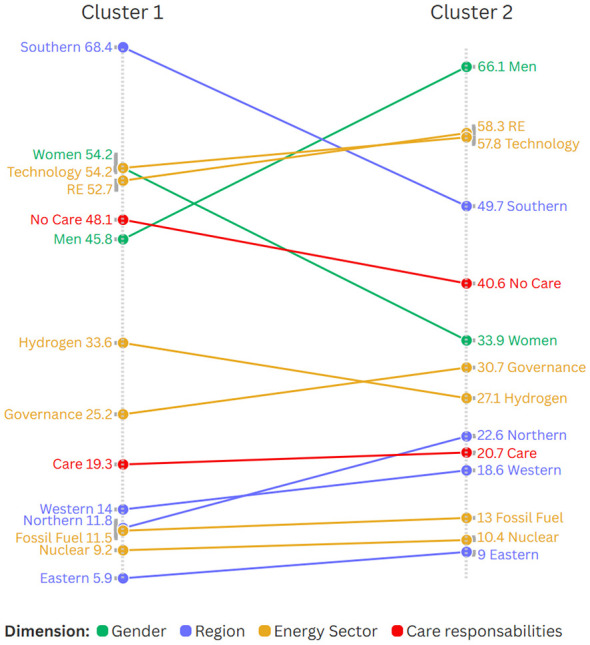
Distribution of the main socio-demographic variables by cluster (%).

The cluster analysis identified two distinct groups of respondents based on nine factors representing different dimensions of the perceived organizational climate. Table 6 provides a comparative analysis of these clusters, using standardized centroid scores to delineate the factor profiles and percentage distributions of key socio-demographic variables. The socio-demographic variables that most effectively characterize each cluster are further illustrated by means of slope chart (Figure 3).

**Table 6:** Comparative cluster.

Dimension	Variable	Cluster 1	Cluster 2
Factors	*WRQoL_F1*	−0.75	0.51
	*WRQoL_F2*	−0.83	0.57
	*WRQoL_F3*	−0.61	0.42
	*PSGBI_F1*	0.69	−0.47
	*PSGBI_F2*	−0.77	0.53
	*PSGBI_F3*	−0.58	0.39
	*PSGBI_F4*	−0.80	0.55
	*JETKP_F1*	0.15	−0.10
	*WDC_F1*	−0.76	0.52
Gender	*Women*	54.2	33.9
	*Men*	45.8	66.1
Age cohorts	22–27	3.8	4.2
	28–35	11.5	13.5
	36–44	25.2	28.6
	45–52	22.9	24.5
	53–60	27.5	17.7
	60+	9.2	11.5
Region	*Southern Europe*	68.4	49.7
	*Northern Europe*	11.8	22.6
	*Western Europe*	14.0	18.6
	*Eastern Europe*	5.9	9.0
Research profile	*Director/Board member*	5.3	5.7
	*Team Manager. Supervisor*	19.1	15.1
	*Researcher or Technologist*	68.7	69.3
Energy sector	*Renewable energy*	52.7	58.3
	*Fossil fuel*	11.5	13.0
	*Hydrogen*	33.6	27.1
	*Nuclear*	9.2	10.4
	*Technological (operational)*	54.2	57.8
	*Governance*	25.2	30.7
Renewable energy technology	*Bioenergy*	19.1	21.9
	*Economic. social and management*	22.9	29.7
	*Geothermal*	19.8	17.2
	*Hydropower*	9.9	10.9
	*Ocean*	10.7	9.9
	*Policy*	16.8	25.5
	*Pumps*	19.8	20.8
	*Solar*	37.4	40.1
	*Waste*	17.6	17.2
	*Wind*	23.7	32.3
Care responsibilities	*Child ≤ 6*	12.2	22.4
	*Child 7–17*	30.5	29.7
	*Elderly*	15.3	9.9
	*No Care*	48.1	40.6

Cluster 1 is characterized by negative values across all factors associated with the *Work-Related Quality of Life* (WRQoL_F), *Perceived Subtle Gender Bias Index* (PSGBI_F), and *Workplace Diversity Climate* (WDC_F) scales. It is important to note that factor PSGBI_F1 exhibits an inverse relationship between score and theoretical meaning: higher values do not indicate a more favorable condition, but rather a higher perception of gender-based discrimination in the workplace.

Overall, respondents in this cluster report markedly negative evaluations across three key dimensions: organizational climate, perceptions of gender discrimination, support, and collegiality in the workplace, and the organizational commitment to promoting diversity and inclusion.

With regard to organizational climate, respondents describe work environments characterized by unclear objectives, which limit the ability to fully express professional potential or to influence change processes within their respective areas of competence (WRQoL_F1). This negative climate is also reflected in limited recognition of work by colleagues, reduced inclusion in decision-making processes, and scarce opportunities for skills development and career advancement (WRQoL_F2). In addition, flexibility tools, such as working hours and agile working arrangements, are perceived as insufficient to support an adequate balance between work demands and family life (WRQoL_F3).

In such organizational contexts, the equity and support practices are severely compromised, fostering a widespread perception of gender discrimination. Respondents report high levels of agreement regarding the presence of discriminatory practices manifested through various forms, including implicit bias, verbal prevarication, exclusion by male colleagues, and differential treatment based solely on gender (PSGBI_F1). These perceptions are further corroborated by negative evaluations of professional relationships, which are described as weakly cohesive and characterized by low levels of mutual support and collaboration (PSGBI_F2). Moreover, a lack of mentoring practices, both peer-based and from senior staff, emerges (PSGBI_F3), indicating work environments that are poorly oriented toward knowledge sharing and professional development. Negative evaluations also extend to PSGBI_F4, which reflects a perceived misalignment between organizational structures and women's professional needs, highlighting insufficient support for work-life balance and the lack of effective policies aimed at promoting equity.

The hostile organizational climate is further reflected in perceptions of the *Workplace Diversity Climate*, with respondents reporting limited organizational commitment to valuing employees with diverse backgrounds, both in recruitment practices and in workforce management and retention strategies. This contributes to a work environment perceived as weakly inclusive and unfavorable to diversity (WDC_F1).

Consistent with these perceptions, respondents in this cluster express high levels of agreement with JETKP_F1, which reflects the perceived need for clearer national and European-level regulations to promote diversity in research and innovation, facilitate labor market entry for underrepresented groups, counteract the male-dominated culture of the energy sector, and implement support policies aimed at making the sector more accessible to minorities.

Given these characteristics, indicative of perceived low job quality and heightened discrimination, we identify Cluster 1 as the *Critical Cluster*.

Cluster 2 comprises respondents who reported generally favorable evaluations across the factors of the same scales, with the exception of PSGBI_F1 and JETKP_F1. As a whole, these assessments delineate an organizational context perceived as more strongly oriented toward work quality and employee needs.

Positive perceptions on the *Work-Related Quality of Life* scale indicate positive levels of professional engagement, clear definition of work objectives, and the ability to influence decision-making processes within one's area of responsibility (WRQoL_F1). Work relationships are predominantly perceived as supportive, characterized by substantial recognition of individual professional contributions and the availability of concrete opportunities for skills development and career advancement (WRQoL_F2). In addition, respondents report employer support for reconciling work and family demands, expressed through flexible working arrangements and schedules aligned with employees' personal circumstances (WRQoL_F3).

Within such work contexts, collegial relationships are also evaluated positively, contributing to an overall climate of mutual appreciation and peer support (PSGBI_F2). Perceptions related to work-life balance are similarly favorable, suggesting strong organizational attention to employees' needs and a concrete commitment to promoting equity (PSGBI_F4). The negative scores observed on PSGBI_F1 indicate that respondents in this cluster tend not to recognize the presence of discriminatory practices or gender stereotypes in their work environments. Consistently, lower levels of agreement are observed for JETKP_F1, which captures support for inclusion policies in the energy sector aimed at promoting cultural diversity, valuing underrepresented groups, and overcoming a predominantly male work culture.

This configuration is partially confirmed by evaluations of WDC_F1, for which a moderate level of agreement emerges, suggesting that respondents perceive their organizational environment as already inclusive and equitable, thereby rendering further institutional intervention unnecessary.

Although Cluster 2 reports overall favorable ratings across the examined factors, it is characterized by scores that remain within moderate thresholds, falling short of high levels of agreement. This intermediate profile, in which perceived satisfaction with the organizational climate does not translate into a critical stance toward structural inequalities or active engagement with diversity and inclusion policies, justifies the designation of the *Neutral Cluster*.

To further characterize the clusters, selected sociodemographic variables were included in the analysis. The observed differences are consistent with the profiles identified (Table 6 and Figure 3).

Gender emerges as the variable with the strongest discriminatory power. In the *Critical Cluster*, women account for 54.2% of respondents, a particularly noteworthy figure given the overall level of dissatisfaction with the factors and the heightened perception of gender inequality (PSGBI_F1). Conversely, men predominate in the *Neutral Cluster* (66.1%), suggesting that work environments perceived as more equitable and inclusive are more frequently experienced by male workers, while women represent only 33.9% of this cluster. The regional distribution also reveals some noteworthy differences across contexts. Although the overrepresentation of Southern Europe in both clusters (*Critical* 68.4%; *Neutral* 49.7%) partly reflects the larger share of respondents from this region, its presence remains significant across both profiles, suggesting that gender-based discrimination is more consistently both perceived and denied within universities and research organizations in this area than in other regions.

With respect to age cohorts, the *Critical Cluster* shows a higher concentration of older workers, particularly within the 53–60 (27.5%), 45–52 (22.9%), and 36–44 (25.2%) age ranges. In contrast, respondents in the *Neutral Cluster* are on average younger, with a higher prevalence in the 36–44 (28.6%) and 45–52 (24.5%) age groups.

Regarding research profile, no substantial differences emerge between clusters. In both groups, approximately 70% of respondents hold positions as Researchers or Technologists, while about 5% occupy Director or Board Member roles. Positions as Team Manager or Supervisor are slightly more frequent in *Critical Cluster* (19.1%) than in *Neutral Cluster* (15.1%).

Considering the energy sector of specialization, some differences in distribution are observed. Respondents in the *Critical Cluster* are more heavily concentrated in Technological operations (54.2%), Renewable Energy (52.7%), and Hydrogen (33.6%). In the *Neutral Cluster*, renewable energy represents a larger share (58.3%), followed by the technological sector (57.8%), alongside a growing presence in Energy Governance (30.7%). In both clusters, fossil fuel and nuclear sectors account for a relatively small proportion of respondents.

To further explore the role of organizations and research institutes in the energy and ecological transition, the analysis also considered renewable energy technologies. In both clusters, respondents are distributed across a similarly broad range of technologies, including Bioenergy, Geothermal, Hydropower, Ocean, and Pumps. Some differences emerge in research activities related to Policy, which are more prevalent in *Neutral Cluster* (8.7%), followed by Wind (8.6%) and Economic, Social and Management research (6.8%).

Finally, care responsibilities reveal particularly meaningful patterns across clusters. In the *Critical Cluster*, a substantial proportion of respondents report not having care responsibilities (48.1%), although significant shares are involved in caring for children aged 7–17 (30.5%) and elderly individuals (15.3%). In line with the negative perceptions, this configuration may reflect difficulties in managing care demands within work environments perceived as insufficiently supportive of employees' individual needs. Given the predominance of women in this cluster, the findings suggest that women may face greater barriers in reconciling professional and family life, potentially leading some to forego additional care responsibilities.

In the *Neutral Cluster*, care responsibilities are more widespread, particularly for children under the age of six (22.4%) and children aged 7–17 (29.7%), while a smaller proportion of respondents report not having care duties (40.6%). This cluster also includes a higher share of younger workers, which is associated with the greater incidence of care especially for preschool-aged children, and is consistent with a work environment perceived as more supportive of work-life balance.

## 5 Discussion

This study aims to provide a descriptive insights into the current European public and private universities and research organizations, with the objective of analyzing the quality of the organizational climate and the extent to which gender equity principles are embedded in institutional practices (Picardi et al., 2023; Wilgosh et al., 2022). The relevance of this inquiry lies in the pivotal role that the energy-related R&I sector plays in the generation of knowledge oriented toward socio-ecological transition. At the same time, the sector continues to be characterized by structural barriers and historical forms of discrimination that systematically affect women and other underrepresented groups. The persistence of a male-dominated culture fosters hostile environments and leaves unresolved the issue of “invisible women” (Smith et al., 2019).

Nevertheless, a corpus of studies cautions that successful climate mitigation efforts cannot be achieved without a talent pool that is inclusive of gender and other forms of diversity (Lazoroska et al., 2024; Hoicka, 2023; Pearl-Martinez and Stephens, 2016), thereby challenging a vision of the energy transition grounded exclusively in technocratic solutions. Despite the higher levels of environmental awareness and the greater propensity for collaborative and inclusive working practices observed among women (Allison et al., 2019), their persistent underrepresentation in the energy sector, including renewables, signals a structural tension between SDG 7 (affordable and clean energy) and SDG 5 (gender equality), the latter being fundamental to ensuring equitable access to employment opportunities within the energy workforce (Ferroukhi et al., 2021).

Our findings empirically confirm this pattern: only 42% of positions in energy research are held by women, compared to 58% by men. Moreover, even with limited sample variability, the data reveal a higher incidence of precarious employment contracts among women, alongside a clear vertical segregation in access to managerial roles within organizations. These outcomes contradict expectations that renewable energy would act as an unheard-of driver for the expansion and diversification of employment opportunities (IRENA, 2019b). The imbalance observed is not incidental, rather it reflects mechanisms of exclusion sustained by interconnected factors. First, misperceptions and the socially constructed nature of gender continue to shape professional roles in line with stereotypical expectations, reproducing enduring patterns of inequality (Vogel et al., 2024; West and Zimmerman, 1987). Such biases, often implicit and unconscious, operate in the early stage of life, influencing women's access to STEM disciplines, which constitute the primary recruitment pipeline for the renewable energy sector (Dunlap and Barth, 2023; Masters and Barth, 2022; Baruah, 2017).

Beyond the numerical representation of women in energy R&I, this study delves into the analysis by examining subjective perceptions of work environments. Cluster analysis reveals a polarization of assessments relating to organizational climate (WRQoL), perceived gender bias (PSGBI), inclusion policies (JETKP) and organizational commitment to diversity valorisation (WDC). Gender emerges as the main explanatory variable. *Critical Cluster*, predominantly female (54.2%), expresses dissatisfaction linked to systemic barriers that not only constrain scientific collaboration, skill development opportunities and career advancement, but also erode the effective chance of participating in decision. Exclusion perceptions from influential networks and stereotypical treatment by male colleagues reinforce feelings of job insecurity (O'Keefe and Courtois, 2019; Sim and Bierema, 2025), hindering professional recognition and workplace respect (Palomba, 2000; Blackwell and Glover, 2008). These findings align with long-standing concerns that male-dominated environments tend to undervalue women's contributions and reinforce gender stereotypes (Etzkowitz et al., 2000; Moss-Racusin et al., 2012), with detrimental consequences both for social equity and for the innovative capacity needed to advance the ecological transition.

A further interesting result emerges from the comparison between research profile and perceived gender bias (PSGBI). Within *Critical Cluster*, women in leadership roles (team manager and/or supervisor) do not seem immune to discrimination. Consistent with existing literature, gender bias and asymmetric evaluations are actually more pronounced for women in senior positions (Lyness and Heilman, 2006). This contrasts with *Neutral Cluster*, predominantly male (66.1%), which converges on positive assessments of organizational climate (WRQoL), does not recognize implicit or explicit forms of gender bias, and views organizational efforts to value professional identities as tangible. This divergence can be interpreted through a dual lens. On the one hand, a form of gender blindness may stem from the benefit of normative advantages or limited direct exposure to discrimination, leading men to overestimate levels of effective equality (García-González et al., 2019). On the other hand, the high concentration of relatively young researchers (53% aged 36–52) predominantly working in renewable energy (58.3%) may signal the emergence of a more dynamic and culturally equity-sensitive environment, potentially capable of overcoming the gender asymmetries historically associated with the traditional energy industry.

Another interpretative axis concerns the interaction between care responsibilities and perceived organizational support. Within the *Critical Cluster*, care burdens related to children (30.5%) and elderly relatives (15.3%) are met with organizational responses perceived as inadequate. However, the same cluster also includes the highest proportion of individuals reporting no care responsibilities (48%), suggesting the presence of more complex and heterogeneous dynamics. These dynamics may include, among others, voluntary or constrained choices, health-related conditions, as well as a reliance on forms of support external to the organization.

In *Neutral Cluster*, despite the presence of care responsibilities for preschool aged children (22.6%) and school-aged children (29.7%), the perceptions of work-life balance measures and organizational wellbeing remain high. The predominance of men within this cluster suggests that such positive assessments may be mediated by lower levels of involvement in domestic labor (Misra et al., 2012; Rosa, 2022), resulting in reduced cognitive investment in negotiating work-life boundaries (Bonache et al., 2022) and a consequent overestimation of the institutional support actually provided. This perception asymmetry deserves further examination, as existing evidence demonstrates that when leave policies are absent or inconsistent, women bear a disproportionate share of the costs, either in the form of reduced working hours or slower career progression (European Commission, Directorate-General for Research and Innovation, 2025).

Moreover, the clusters delineate contrasting views of institutional commitment to diversity and inclusion practices (WDC). Respondents in *Critical Cluster* perceive limited organizational engagement both in recruitment processes and in retention strategies for a diverse workforce, suggesting that inclusion often remains a formal exercise, lacking translation into established practices. As observed in other research contexts, women more frequently perceive their professional environments as structurally less inclusive (Pinho and Colston, 2024) and are more inclined to identify and report gender discrimination, particularly in male-dominated sectors (Parker, 2018).

In line with this evaluation, *Critical Cluster* expresses support for the policy advocacy for inclusivity in the energy sector (JETKP), emphasizing the urgency of clear national and European regulatory frameworks to promote diversity in R&I, facilitate access to the labor market for underrepresented groups, and actively challenge the male-dominated culture of the energy sector through targeted support measure. By contrast, respondents in *Neutral Cluster*, who perceive existing workplace as already inclusive, display lower support for the introduction of inclusive policies. This suggests a privilege bias: those who experience the organization as fair tend to view corrective regulatory interventions as unnecessary or excessive, as they believe that gender and other forms of social equalities have been reached (Brannon et al., 2018).

Further analytical element concerns the geographical distribution of respondents. The data reveal a notable regional variation: Southern Europe is overrepresented in the *Critical Cluster* (68.4%) compared to the *Neutral Cluster* (49.7%), while Northern (11.8% vs. 22.6%), Western (14% vs. 18.6%), and Eastern Europe (5.9% vs. 9%) show higher shares in the *Neutral Cluster*. This pattern may reflect differences in institutional contexts and organizational cultures, with Southern Europe historically characterized by less consolidated normative frameworks on workplace gender equality (Farina et al., 2023). While these findings do not allow for generalizations, they suggest that gender inequalities in energy-related R&I may have a geographical dimension that warrants attention in the design of contextually differentiated policy interventions.

Overall, perceptions analysis point to the inadequacy of existing employment equality policies. Many of the measures in place often fall short, as they neither address the complexity of institutional factors constraining women's careers nor promote recruitment practices that can correct entrenched historical asymmetries (Baruah, 2017). Energy policy, which remains largely blind to gender issues, thus fails to dismantle structural barriers to equality (Clancy, 2025), confining the energy transition within limits that risk reproducing the same inequalities of pre-existing fossil fuel-based systems.

### 5.1 Limitations

This study acknowledges certain methodological shortcomings, which nonetheless offer insights for future research on the workforce in the R&I sector. First, although the survey was distributed across public and private research institutions in multiple European countries, private companies were underrepresented, limiting the inclusion of their perspectives. Given the crucial role of these organizations in energy research, future studies should adopt targeted strategies to ensure balanced representation and capture both perceptions of the internal workplace climate and gender dynamics. A second limit is the sampling: using snowball methods limited internal variability, with low participation from non-permanent research profiles, such as PhD candidates and postdoctoral fellows, who, despite the precarious nature of their employment, contribute significantly to scientific and technological developments in the sector. Enhancing sample diversity could strengthen data robustness and enable more nuanced and generalizable analyses. Beyond its analytical scope, the survey also served a reflection tool for organizations, helping to foster inclusive, gender-sensitive, and collaborative environments. However, while some stakeholders fully leveraged its potential, others did not use the findings for internal evaluation and organizational commitment. Future research could implement more targeted communication strategies, highlighting the dual benefit of participation: contributing to advancing research knowledge and obtaining usable internal information. Due to these limitations, the findings primarily provide indications of perceptions of the workplace climate within universities and research organizations, rather than statements generalizable to the entire sector.

### 5.2 Conclusion

To what extent do universities and research organizations promote gender inclusion and recognize professional identities? To address this question, the present study analyzed the perceptions of R&I workforce in the energy sector, contributing to the scholarly literature that cautions against dismissing the gender issue in the ecological transition to a merely quantitative matter. The role of European academia and research organizations was examined through two analytical lenses: on the one hand, support for women's inclusion in career pathways and the removal of gender asymmetries; on the other, organizational practices centered on professional recognition, empowerment and workplace wellbeing.

The findings provide a nuanced picture of the experiences of the workforce based on the respondents' perceptions cluster into two distinct groups, from which insights emerge that are relevant not only for advancing scientific knowledge but also for informing policy recommendations.

The results offer a nuanced account of workforce experiences, with respondents' perceptions falling into two distinct clusters that yield insights relevant to both advancing scientific knowledge and informing policy recommendations.

The *Critical Cluster* highlights how gender inequalities are structurally rooted in universities and research organizations in the energy sector. These dynamics negatively affect the quality of working environments, manifesting through discriminatory practices, microaggressions, verbal harassment and the systematic undervaluation of women's professional contributions, as well as through the perception of limited organizational commitment to diversity and inclusion. Persistent penalties further emerge regarding both career opportunities and the reconciliation of work and care responsibilities. From this perspective, a future line of research could explore the extent to which the workplace climate in energy R&I influences intentions toward parenthood, particularly among women, thereby shedding light on the role of organizational constraints in shaping life choices and professional trajectories. The evidence thus points to the need to strengthen existing equality measures, moving beyond a purely procedural implementation of Gender Equality Plans and promoting, at the same time, rigorous monitoring mechanisms alongside organizational reforms aimed at removing the structural barriers.

The *Neutral Cluster* presents a more ambivalent picture that warrants further analytical scrutiny. Respondents, predominantly men, perceive their working environments as inclusive and fair, characterized by work-life balance measures, career opportunities and adequate collegial support, while failing to recognize the presence of gender bias. Moreover, these organizations are perceived as being oriented toward the broader valorisation of diversity. A substantial share of respondents in this cluster work in the renewable energy sector, which may have internalized, at least at a discursive level, principles of sustainability, equity and social justice. However, the available evidence does not allow us to determine accurately whether these perceptions are primarily shaped by respondents' gender or by sector-specific characteristics, thereby paving the way for future research.

Nevertheless, the latter cluster raises two major concerns. First, it suggests that even within the renewable sector commonly perceived as innovative and dynamic, gender barriers persist and remain insufficiently recognized. Second, the gender blindness may reflect omission biases typical of dominant groups, who tend to view more equitable and inclusive policies as unnecessary in contexts perceived as already neutral. While these findings should be interpreted with caution, they flag delays in the transformation of organizational cultures, which continue to reproduce exclusionary power relations and to underestimate the contribution that diverse professional identities can provide to the imperatives of climate change mitigation.

Addressing these unresolved issues will require a systemic and multi-level approach, grounded in institutional reforms, targeted mentoring programmes and proactive diversity policies. In this respect, a model based exclusively on a formal conception of meritocracy has proven insufficient to ensure equitable access to research and inclusive career progression. From this perspective, achieving gender equality in energy-related R&I constitutes a strategic priority, not only to harness the full spectrum of available talents and competences, but also to integrate diverse perspectives into energy research agendas. This would help ensure that the ecological transition is not only technologically advanced, but also socially equitable and just.

### 5.3 Policy recommendations

Our finding provides empirical evidence that gender inequalities in energy-related R&I are sustained not only by patterns of underrepresentation, but also by organizational cultures and everyday practices that remain largely resistant to change. Such dynamics have direct implications for the governance of energy transitions, as they shape who produces knowledge, which perspectives are legitimized, and how innovation trajectories are defined. From this perspective, gender equality in R&I emerges as a structural dimension of a just and effective energy transition. Our study suggests four interrelated policy implications for universities and research organizations engaged in energy-related R&I: i) reframing Gender Equality Plans as instruments of organizational transformation; ii) embedding organizational climate and inclusion within R&I governance frameworks; iii) situating work-life balance within the political economy of energy research and finally iv) linking organizational inequalities to the justice dimension of energy transitions.

i) Our findings suggest that Gender Equality Plans (GEPs), while increasingly widespread across European research institutions, often struggle to translate formal commitments into substantive organizational change. Rather than functioning primarily as compliance mechanisms, GEPs should be understood as reflexive governance tools, capable of addressing informal norms, power relations, and career structures within research organizations. Greater attention to qualitative indicators, such as perceptions of recognition, inclusion, and fairness, would allow for a more nuanced assessment of institutional change.
ii) Current R&I governance frameworks continue to prioritize output-based indicators of excellence, with limited attention to the organizational conditions under which research is produced. The results indicate that workplace climate, access to mentoring, and perceived gender bias are central to researchers' experiences and retention. Integrating these dimensions into evaluation and funding criteria could contribute to aligning research governance with broader sustainability and justice objectives.
iii) Work-life balance measures emerge as a critical dimension of organizational wellbeing, yet their availability and legitimacy vary widely across institutional contexts. The findings suggest that such measures should be analyzed not as individual accommodations, but as structural features of research systems that influence who can remain and progress within energy R&I. Addressing work-life balance is therefore integral to sustaining a diverse and stable research workforce.
iv) The persistence of gendered organizational barriers raises broader questions about the social foundations of the energy transition. If research institutions reproduce exclusionary practices, the risk is that innovation pathways will continue to reflect narrow social perspectives. Integrating gender and diversity considerations into energy of R&I governance is thus not only a matter of equity, but a condition for producing socially robust and legitimate knowledge for low-carbon transitions.

## Data Availability

The datasets presented in this study can be found in online repositories. The names of the repository/repositories and accession number(s) can be found below: https://doi.org/10.5281/zenodo.18257325.
