# Supplementary material for: Hostile work climate: gender barriers in the European energy R&I workforce
Source: Front Sociol. 2026 Jun 3;11:1800176. doi: 10.3389/fsoc.2026.1800176 (PMC13271930; doi:10.3389/fsoc.2026.1800176)
Supplement: Supplementary file 1 [file Supplementary_File_1.docx]

Supplementary Material

**Annex 1 – Methodology full description**

# Introduction

The objective of the factor analysis was to reduce the complexity of a survey structured around multiple question batteries by identifying and validating underlying factorial structures through Exploratory Factor Analysis (EFA) and Confirmatory Factor Analysis (CFA).

The analysis has been performed on the following psychometric scales:

Work-Related Quality of Life (WRQoL). Adapted from Easton and Van Laar (2018)^[[1]](#footnote-1)^, this scale measures the perceived quality of life of employees (12 questions).

Perceived Subtle Gender Bias Index (PSGBI). Elaborated by Tran et al. (2019)^[[2]](#footnote-2)^, the PSGBI measures the perceptions that an individual holds regarding subtle experiences that are related to their gender identification (21 questions).

Perspective for just energy transition knowledge production (JETKP). We created this scale to grasp individuals’ thoughts regarding what type of policies need to be adopted to make the energy sector more inclusive (6 questions).

Workplace Diversity Climate (WDC). Adapted from Ward et al. (2022)^[[3]](#footnote-3)^, this scale measure individual’s perceptions about the diversity climate in their workplaces (4 questions).

For the PSGBI we proceeded with a Confirmatory Factor Analysis (CFA), and the latent factors have been developed based on Tran et al. (2019). Since we used the full set of questions, in fact, we decided to code the latent factor as illustrated by the authors. On the contrary, for WRQoL, WDC, and JETKP, since the first and the second have been adapted from already available scales and the third has been appositely created for this survey, before proceeding with the CFA we performed an Exploratory Factor Analysis (EFA). As discussed by Suhr (2006)^[[4]](#footnote-4)^ the EFA is employed to explore the underlying factor structure of the data and to identify the number of latent constructs in the variables’ set.

The factors obtained from CFA were detected with Cluster Analysis in order to identify latent profiles within the R&I workforce of the energy sector.

Below, the methodological steps undertaken in each phase are described.

Supplementary material is not typeset so please ensure that all information is clearly presented, the appropriate caption is included in the file and not in the manuscript, and that the style conforms to the rest of the article.

# Data Preparation

## Transformation of Likert scale variables into numerical variables

For the questions’ batteries WRQoL, PSGB, JETKP and WDC, survey responses, initially in textual format (Agree strongly, Agree, Neither disagree nor agree, Disagree, Disagree strongly), were recoded into numerical values (1=Disagree strongly – 5=Agree strongly).

Missing responses ("No answer") were treated as missing values (NA) to ensure the accuracy of subsequent analyses.

## Recoding other variables

The variable Seniority, originally coded as categorical (Junior (0-4), Middle career (5-7), Senior (7-x)), has been recoded into an ordinal scale (1 = Junior (0-4), 2 = Middle career (5-7), 3 = Senior (7-x)).

The variable Organization_size, originally coded as categorical (1 to 9, 10 to 49, 50 to 249, 250 or more), has been recoded into an ordinal scale (1 = 1 to 9, 2 = 10 to 49, 3 = 50 to 249, 4 = 250 or more).

The variable Education, originally coded as categorical (High School (ISCED 3), Bachelor's degree (ISCED 6), Professional degree (e.g., law or medicine) (ISCED 6A/7), Master's degree or equivalent (ISCED 7), Doctorate degree (ISCED 8)), has been recoded into an ordinal scale (1 = High School (ISCED 3), 2 = Bachelor's degree (ISCED 6), 3 = Professional degree (e.g., law or medicine) (ISCED 6A/7), 4 = Master's degree or equivalent (ISCED 7), 5 = Doctorate degree (ISCED 8)).

Country is a nominal variable coded according to the Geographic Regions scheme of the United Nations – Statistics Division. Countries are grouped into four European macro-regions: Eastern Europe (Poland, Romania, Bulgaria); Northern Europe (United Kingdom, Denmark, Finland, Sweden, Ireland, Norway); Southern Europe (Spain, Italy, Portugal, Greece, Slovenia, Croatia); and Western Europe (Germany, Belgium, France, Austria, Netherlands).

## Sociodemographic variables by gender

Below, Table 1 shows the percentage distribution by gender (women and men) of key sociodemographic variables, offering a general overview of the respondents' profiles.

*Table 1: Sociodemographic variables by gender*

| Variable | Category | Women | Men |
| --- | --- | --- | --- |
| *Gender* | Male | - | 57,9 |
|  | Female | 42,1 | - |
| *Age Group* | >70 | 0,7 | 1,6 |
|  | 66-70 | 4,4 | 2,3 |
|  | 61-65 | 12,5 | 10,2 |
|  | 56-60 | 16,9 | 14,4 |
|  | 51-55 | 16,1 | 15,5 |
|  | 46-50 | 16,1 | 10,2 |
|  | 41-45 | 14,7 | 16,6 |
|  | 36-40 | 11,8 | 15,5 |
|  | 31-35 | 10,3 | 6,9 |
|  | 26-30 | 6,6 | 6,4 |
|  | 22-25 | 4,4 | 0 |
| *Seniority* | Senior (7+ years) | 67,6 | 79,1 |
|  | Middle career (5-7 years) | 13,9 | 10,1 |
|  | Junior (0-4 years) | 18,3 | 10.7 |
| *Research profile* | Researcher or Technologist | 69,1 | 68,9 |
|  | Team Manager/Supervisor | 16,1 | 17,1 |
|  | Director/Board Member | 4,4 | 6,4 |
|  | Technician | 0,7 | 3,2 |
|  | Research Assistant | 5,9 | 1,6 |
|  | Other | 3,6 | 2,6 |
| *Care Responsibilities* | None | 41,9 | 44,9 |
|  | Elderly | 14,7 | 10,1 |
|  | Disability | 2,2 | 5,4 |
|  | Children 7-17 | 29,4 | 30,5 |
|  | Children under 6 | 18,3 | 18,2 |
| *Education Level* | Doctorate (ISCED 8) | 67,8 | 71,4 |
|  | Master's (ISCED 7) | 25,9 | 22,1 |
|  | Bachelor's (ISCED 6) | 4,9 | 1,5 |
|  | Professional degree (ISCED 6A/7) | 0,7 | 3,5 |
|  | High School (ISCED 3) | 0,7 | 1,5 |
| *Employment Type* | Public and Private Academic/Research Organization | 91,9 | 93 |
|  | Private Company/Corporation | 4,4 | 4,3 |
|  | Self-employed (Academic/Research) | 1,5 | 1,6 |
|  | Self-employed (Business) | 2,2 | 1 |
| *Contract Type* | Permanent position contract | 75,6 | 80,7 |
|  | Fixed term contract | 17,6 | 13,7 |
|  | Apprenticeship/Training (PhD, scholarship, internships) | 5,3 | 3,3 |
|  | Temporary employment agency contract | 1,5 | 2,2 |
| *Energy Sector* | Coal | 0,7 | 5,6 |
|  | Energy efficiency | 34,6 | 43,3 |
|  | Finance | 4,4 | 6,4 |
|  | HVAC | 2,9 | 11,8 |
|  | Hydrogen | 21,3 | 35,8 |
|  | Nuclear | 9,6 | 10,1 |
|  | Oil & Gas | 8,9 | 11,8 |
|  | Other | 6,6 | 5,6 |
|  | Policy | 20,6 | 20,3 |
|  | Renewable Energy | 51,5 | 59,4 |
|  | Regulation | 13,8 | 16,6 |
|  | Retail | 2,2 | 4,3 |
|  | Storage | 21,3 | 34,2 |
|  | Transmission & Distribution | 9,6 | 18,7 |
| *Renewable Energy Technology* | Bioenergy | 17,7 | 22,3 |
|  | Ecosocman | 19,6 | 32,1 |
|  | Geothermal | 13,8 | 21,4 |
|  | Hydropower | 8,1 | 12,3 |
|  | Ocean | 9,6 | 10,7 |
|  | Other | 8,1 | 8 |
|  | Policy | 16,9 | 25,7 |
|  | Pumps | 16,2 | 23,5 |
|  | Solar | 33,1 | 43,3 |
|  | Waste | 13,4 | 19,8 |
|  | Wind | 19,6 | 35,3 |

# Exploratory Factor Analysis (EFA)

EFA was used to explore the latent factorial structures underlying the three question batteries WRQoL, WDC, and JETKP, and to identify meaningful dimensions.

## WRQoL (Work-Related Quality of Life)

Twelve variables related to work-related quality of life were included after being numerically recoded:

WRQoL_influence_N; WRQoL_abilities_N; WRQoL_goals_N; WRQoL_aknowledgement_N; WRQoL_skill_development_N; WRQoL_pressure_N; WRQoL_decision_involvement_N; WRQoL_career_opportunity_N; WRQoL_needs_met_N; WRQoL_safe_environment_N; WRQoL_working_hours_N; WRQoL_flexibility_N

The Bartlett’s test of sphericity and KMO (Kaiser-Meyer-Olkin) measure confirmed the adequacy of the correlation matrix for factor analysis. The Bartlett’s test shows a chi-square value of 1501.411 degrees of freedom and a P value of 0.00. The Kaiser-Meyer-Olkin (KMO) test shows an Overall Measure of Sampling Adequacy (MSA): of 0.87. Item-level MSAs range from 0.75 to 0.93, but WRQoL_pressure_N has a low MSA (0.56).

The Kaiser criterion identifies the presence of 3 Factors (eigenvalues > 1): Factor 1: 4.93, Factor 2: 1.26, and Factor 3: 1.23. The Parallel analysis (Figure 1) further validated the selection of three factors.

*Figure 1: Parallel analysis for WRQoL*

*
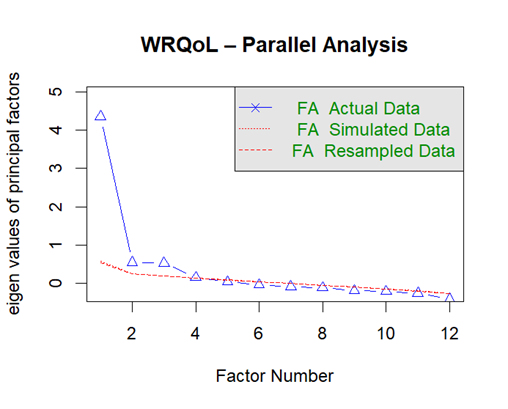
*

Factors were extracted using the Maximum Likelihood (ML) method with Varimax rotation for improved interpretability. The diagram in Figure 2 shows the factor loadings for all the variables.

*Figure 2: Factors extracted for WRQoL and relative factor loadings*


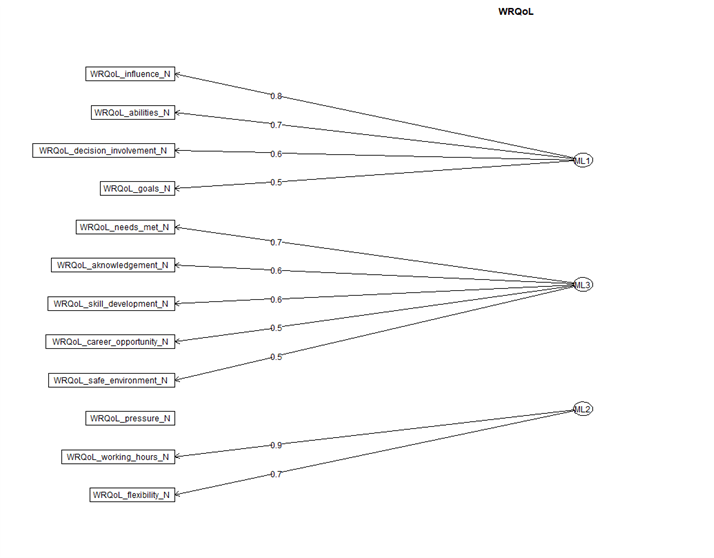


Model fit statistics: RMSR = 0.03; RMSEA = 0.062; Chi-square = 75.35 with p < 0.000; TLI = 0.941; BIC = -116.42. Factor Score Adequacy: ML3 (correlation = 0.85, R² = 0.72, minimum correlation = 0.44); ML2 (correlation = 0.90, R² = 0.81, minimum correlation = 0.61); ML1 (correlation = 0.87, R² = 0.75, minimum correlation = 0.50).

## Perspective for just energy transition knowledge production (JETKP)

Six variables related to JETKP were included after being numerically recoded: Policy_diversity_N; Policy_culture_N; Policy_favoring_groups_N; Policy_society_representation_N; Policy_male_domination_N; Policy_minorities_N.

The Bartlett’s test of sphericity and KMO (Kaiser-Meyer-Olkin) measure confirmed the adequacy of the correlation matrix for factor analysis. The Bartlett’s test shows a chi-square value of 809.108, 15 degrees of freedom and a P value of 0.00. The Kaiser-Meyer-Olkin (KMO) test shows an Overall Measure of Sampling Adequacy (MSA): of 0.86. Item-level MSAs range from 0.82 to 0.89.

The Kaiser criterion identifies the presence of 3 Factors (eigenvalues > 1): Factor 1: 3.52. The Parallel analysis further validated the selection of one factor (Figure 3).

*Figure 3: Parallel analysis for JETKP*

*
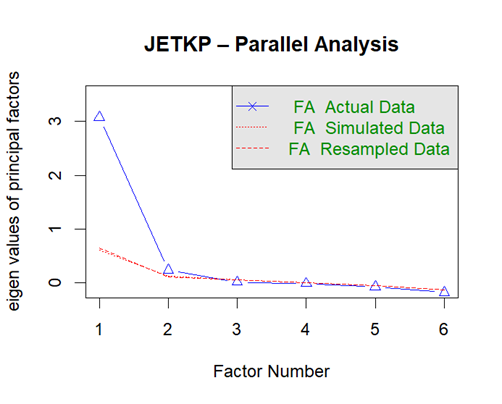
*

Factors were extracted using the Maximum Likelihood (ML) method with Varimax rotation for improved interpretability. The diagram in Figure 4 shows the factor loadings for all the variables.

*Figure 4: Factors extracted for JETKP and relative factor loadings*


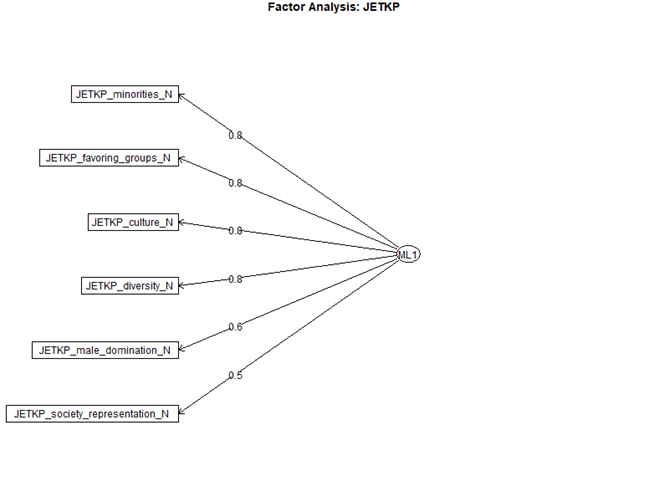


Model fit statistics: RMSR = 0.06; RMSEA = 0.12; Chi-square = 49.76 with p < 0.000; TLI = 0.914; BIC = -1.98. Factor Score Adequacy: ML1 (correlation = 0.94, R² = 0.88, minimum correlation = 0.76).

## Workplace Diversity Climate (WDC)

Four variables related to WDC were included after being numerically recoded: Organization_managing_backgrounds_N; Organization_accepted_backgrounds_N; Organization_hiring_practices_N; Organization_retain_diversity_N.

The Bartlett’s test of sphericity and KMO (Kaiser-Meyer-Olkin) measure confirmed the adequacy of the correlation matrix for factor analysis. The Bartlett’s test shows a chi-square value of 553.682, 6 degrees of freedom and a P value of 0.00. The Kaiser-Meyer-Olkin (KMO) test shows an Overall Measure of Sampling Adequacy (MSA): of 0.81. Item-level MSAs range from 0.78 to 0.85.

The Kaiser criterion identifies the presence of 3 Factors (eigenvalues > 1): Factor 1: 2.814. The Parallel analysis further validated the selection of one factor (Figure 5).

*Figure 5: Parallel analysis for WDC*

*
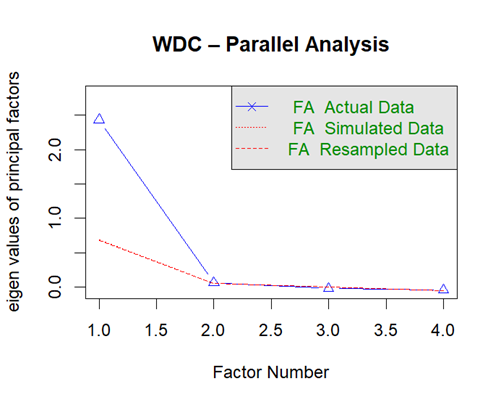
*

Factors were extracted using the Maximum Likelihood (ML) method with Varimax rotation for improved interpretability. The diagram in Figure 6 shows the factor loadings for all the variables.

*Figure 6: Factors extracted for WDC and relative factor loadings*

*
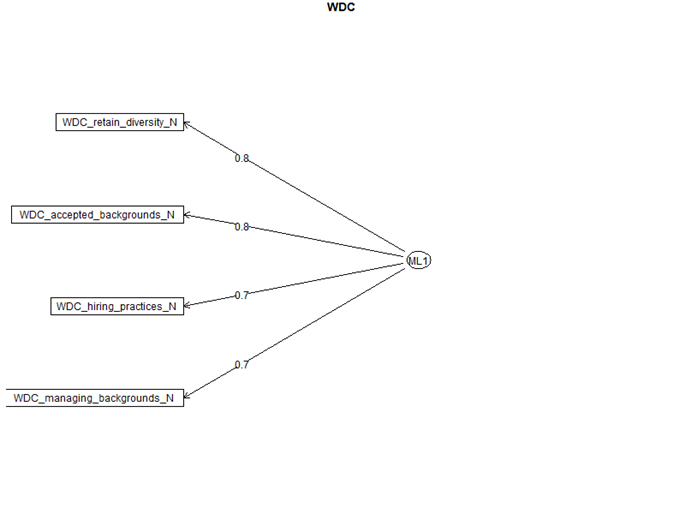
*

Model fit statistics: RMSR = 0.02; RMSEA = 0.08; Chi-square = 6.01 with p < 0.000; TLI = 0.978; BIC = -5.46. Factor Score Adequacy: ML1 (correlation = 0.93, R² = 0.87, minimum correlation = 0.74).

# Confirmatory Factor Analysis (CFA)

## Perceived Subtle Gender Bias Index (*PSGBI*)

The model specified included four latent factors and was composed as shown in Figure 7 below.

*Figure 7: PSGBI Factors composition*


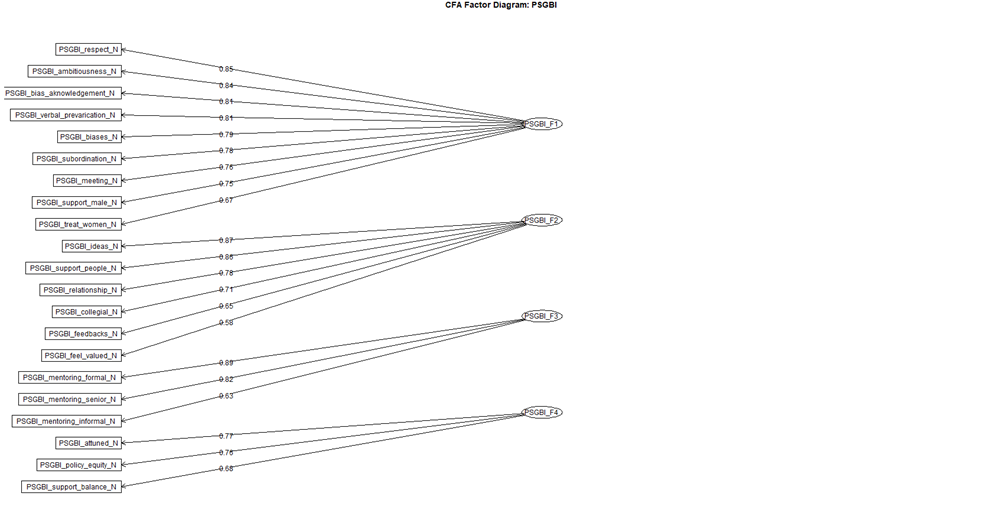


Parameter estimates were obtained using the robust Maximum Likelihood Estimation method (MLR) with missing data handled using the "ML" option. Model fit indices (e.g., CFI, TLI, RMSEA, SRMR) were used to evaluate the goodness-of-fit of the models and are reported in table 2. Acceptable index values confirmed the validity of the proposed factorial structure.

*Table 2: Model fit indicators PSGBI CFA*

| **Statistic** | **Value** |
| --- | --- |
| Chi-Square Test Statistic | 464.412 |
| Degrees of Freedom | 183 |
| P-value (Chi-square) | <0.001 |
| Comparative Fit Index (CFI) | 0.931 |
| Tucker-Lewis Index (TLI) | 0.920 |
| Root Mean Square Error of Approximation (RMSEA) | 0.066 |
| RMSEA 90% CI (Lower) | 0.058 |
| RMSEA 90% CI (Upper) | 0.073 |
| Standardized Root Mean Square Residual (SRMR) | 0.057 |
| Bayesian Information Criterion (BIC) | 18201.79 |

Factor scores were computed for each validated CFA model and added to the dataset as new variables. This allowed the latent dimensions to be represented synthetically, reducing the number of variables in the original dataset. The four factors, PSGBI_F1, PSGBI_F2,PSGBI_F3, and PSGBI_F4, respectively identified the following constructs: Perceptions of Gender-Based Inequality; Perceived Workplace Support and Collegiality; Mentorship and Professional Guidance; and Support and Work-Life Balance.

## Work-Related Quality of Life (WRQoL)

The model specified included four latent factors and was composed as reported in Figure 8.

*Figure 8: WRQoL Factors composition.*

*
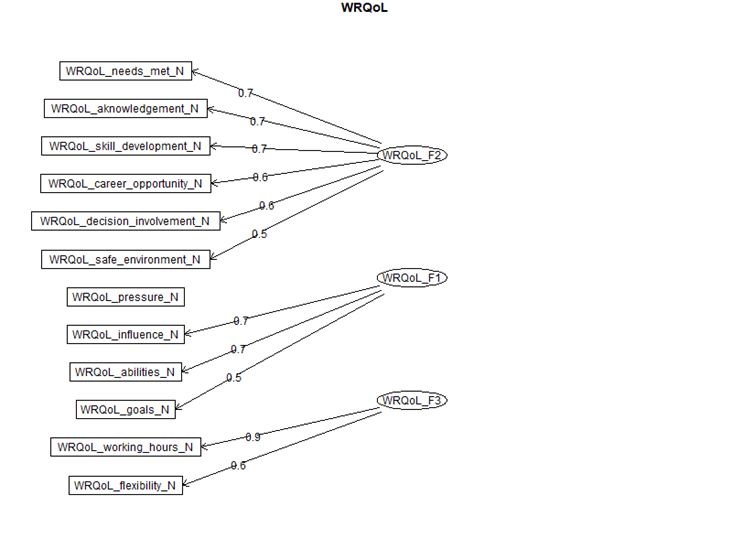
*

Parameter estimates were obtained using the robust Maximum Likelihood Estimation method (MLR) with missing data handled using the "ML" option. Model fit indices (e.g., CFI, TLI, RMSEA, SRMR) were used to evaluate the goodness-of-fit of the models and are reported in Table 3. Acceptable index values confirmed the validity of the proposed factorial structure.

*Table 3: Model fit indicators WRQoL CFA*

| **Statistic** | **Value** |
| --- | --- |
| Chi-Square Test Statistic | 125.936 |
| Degrees of Freedom | 41 |
| P-value (Chi-square) | 0 |
| Comparative Fit Index (CFI) | 0.944 |
| Tucker-Lewis Index (TLI) | 0.925 |
| Root Mean Square Error of Approximation (RMSEA) | 0.076 |
| RMSEA 90% CI (Lower) | 0.061 |
| RMSEA 90% CI (Upper) | 0.092 |
| Standardized Root Mean Square Residual (SRMR) | 0.043 |
| Bayesian Information Criterion (BIC) | 9824.600 |

Factor scores were computed for each validated CFA model and added to the dataset as new variables. This allowed the latent dimensions to be represented synthetically, reducing the number of variables in the original dataset. The three factors, WRQoL_F1, WRQoL_F2, WRQoL_F3, respectively identified the following constructs: Empowerment and Goal Clarity in the Workplace; Workplace Support and Professional Development Opportunities; and Work-Life balance and Flexibility.

## Perspective for just energy transition knowledge production (JETKP)

The model specified included four latent factors and was composed as reported in Figure 9.

*Figure 9: JETPK Factors composition*

*
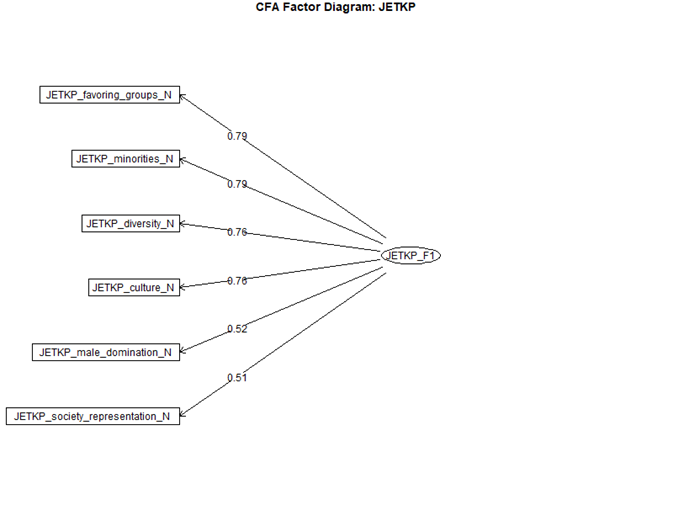
*

Parameter estimates were obtained using the robust Maximum Likelihood Estimation method (MLR) with missing data handled using the "ML" option. Model fit indices (e.g., CFI, TLI, RMSEA, SRMR) were used to evaluate the goodness-of-fit of the models and are reported in Table 4. Acceptable index values confirmed the validity of the proposed factorial structure.

*Table 4: Model fit indicators JETPK CFA*

| **Statistic** | **Value** |
| --- | --- |
| Chi-Square Test Statistic | 58.939 |
| Degrees of Freedom | 9 |
| P-value (Chi-square) | 0.000 |
| Comparative Fit Index (CFI) | 0.936 |
| Tucker-Lewis Index (TLI) | 0.893 |
| Root Mean Square Error of Approximation (RMSEA) | 0.128 |
| RMSEA 90% CI (Lower) | 0.098 |
| RMSEA 90% CI (Upper) | 0.160 |
| Standardized Root Mean Square Residual (SRMR) | 0.045 |
| Bayesian Information Criterion (BIC) | 5052.628 |

Factor scores were computed for each validated CFA model and added to the dataset as new variables. This allowed the latent dimensions to be represented synthetically, reducing the number of variables in the original dataset. The one factor, JETKP _F1, identified the following construct: Policy Advocacy for Inclusivity in the Energy Sector.

## Workplace Diversity Climate (WDC)

The model specified included four latent factors and was composed as reported in Figure 10.

*Figure 10: WDC Factors composition*

*
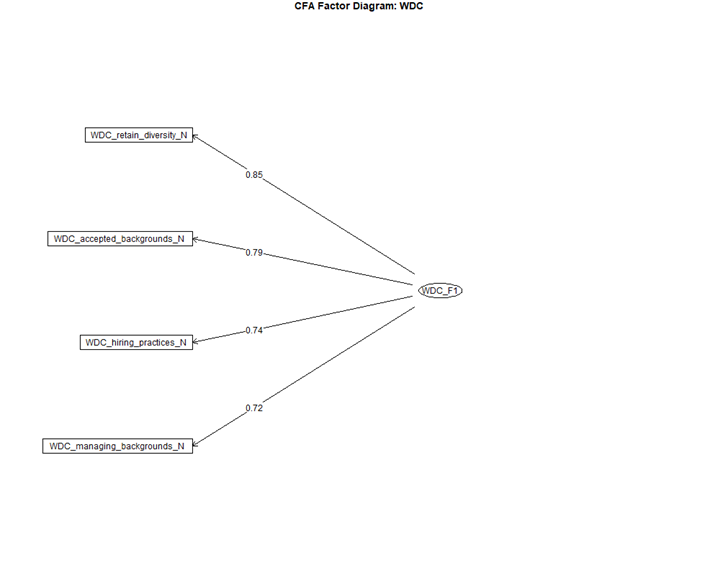
*

Parameter estimates were obtained using the robust Maximum Likelihood Estimation method (MLR) with missing data handled using the "ML" option. Model fit indices (e.g., CFI, TLI, RMSEA, SRMR) were used to evaluate the goodness-of-fit of the models and are reported in Table 5. Acceptable index values confirmed the validity of the proposed factorial structure.

*Table 5: Model fit indicators WDC CFA*

| **Statistic** | **Value** |
| --- | --- |
| Chi-Square Test Statistic | 567.164 |
| Degrees of Freedom | 6 |
| P-value (Chi-square) | 0.000 |
| Comparative Fit Index (CFI) | 0.994 |
| Tucker-Lewis Index (TLI) | 0.983 |
| Root Mean Square Error of Approximation (RMSEA) | 0.068 |
| RMSEA 90% CI (Lower) | 0.000 |
| RMSEA 90% CI (Upper) | 0.144 |
| Standardized Root Mean Square Residual (SRMR) | 0.014 |
| Bayesian Information Criterion (BIC) | 3425.547 |

Factor scores were computed for each validated CFA model and added to the dataset as new variables. This allowed the latent dimensions to be represented synthetically, reducing the number of variables in the original dataset. The one factor, WDC_F1, identified the following construct: Commitment to Diversity and Inclusion.

## Description of the constructs of the extracted factors

Table 6 provides an overview of the factors extracted for each set of questions and the corresponding construct they represent.

*Table 6: List of the extracted factors and the relative construct identified*

| Extracted factor | Construct | Construct description |
| --- | --- | --- |
| WRQoL_F1 | Work Self-Efficacy | The construct reflects an individual’s sense of autonomy, purpose, and meaningful contribution in their professional role. It captures employees’ clarity of objectives, effective skills utilization, and freedom to express opinions and influence decisions, emphasizing a workplace that fosters personal agency, and alignment with organizational goals |
| WRQoL_F2 | Support and Recognition | The construct assesses employees' perceived support and recognition at work, including achievement recognition, skill development, decision-making involvement, resource access, and workplace safety. It also reflects career growth satisfaction, emphasizing an organizational culture that prioritizes professional development and well-being. |
| WRQoL_F3 | Organizational Flexibility | The construct assesses the extent to which the workplace supports work-life balance through flexible working hours and adaptable arrangements, enabling employees to manage professional and personal responsibilities and promoting a healthy work-life balance. |
| PSGBI_F1 | Gender Bias | The construct assesses perceptions of gender bias in communication, respect, recognition, and support for challenges specific to women. It reflects awareness of disparities in opportunities and treatment between male and female colleagues, including both subtle and overt workplace inequities. |
| PSGBI_F2 | Workplace cohesion | This construct reflects workplace support and recognition, encompassing collegiality, appreciation, and inclusivity. It captures employees' sense of value, positive feedback on their abilities, and supportive relationships, emphasizing a collaborative and respectful work culture. |
| PSGBI_F3 | Mentorship and Professional Guidance | The construct captures both informal and formal mentoring experiences, including one-on-one interactions and connections with senior leadership mentors, emphasizing the role of mentorship in fostering career growth and development. |
| PSGBI_F4 | Equity-Driven Climate | The construct reflects the perception of organizational efforts to create an equitable and inclusive workplace. It captures how well the organization recognizes and supports the professional needs of employees, provides resources for balancing work and family responsibilities, and upholds policies that promote fairness and equity. |
| JETKP_F1 | Policy Advocacy for Inclusivity in the Energy Sector | The construct reflects support for policies and systemic changes promoting greater diversity and inclusion in the energy sector. This implies the need for clear regulations, cultural shifts, and government intervention to create an equitable workforce, advocating for dismantling existing barriers and fostering inclusivity for the benefit of underrepresented groups. |
| WDC_F1 | Diversity and Inclusion Practices | The construct represents an organization's commitment to equity and inclusion, encompassing fair hiring, diversity management, and a culture of acceptance. It also highlights leadership's role in fostering a diverse workforce through equitable actions and inclusive policies. |

# Cluster Analysis

Cluster analysis was performed on the nine factors obtained through CFA to identify latent profiles within the R&I workforce of the energy sector.

The procedure was structured into four sequential steps. First, the factor scores derived from the CFA were standardized (*z-scores*). This pre-processing step was essential to normalise the measurement scales across all dimensions, ensuring that each factor contributed with equal metric weight to the distance computation.

Subsequently, the optimal number of clusters (K) was determined through the application of the Elbow Method (Figure 11) and the Gap Statistic (Figure 12). Both criteria provided consistent evidence, suggesting a two-cluster solution as the most parsimonious compromise between model complexity and descriptive capacity.

*
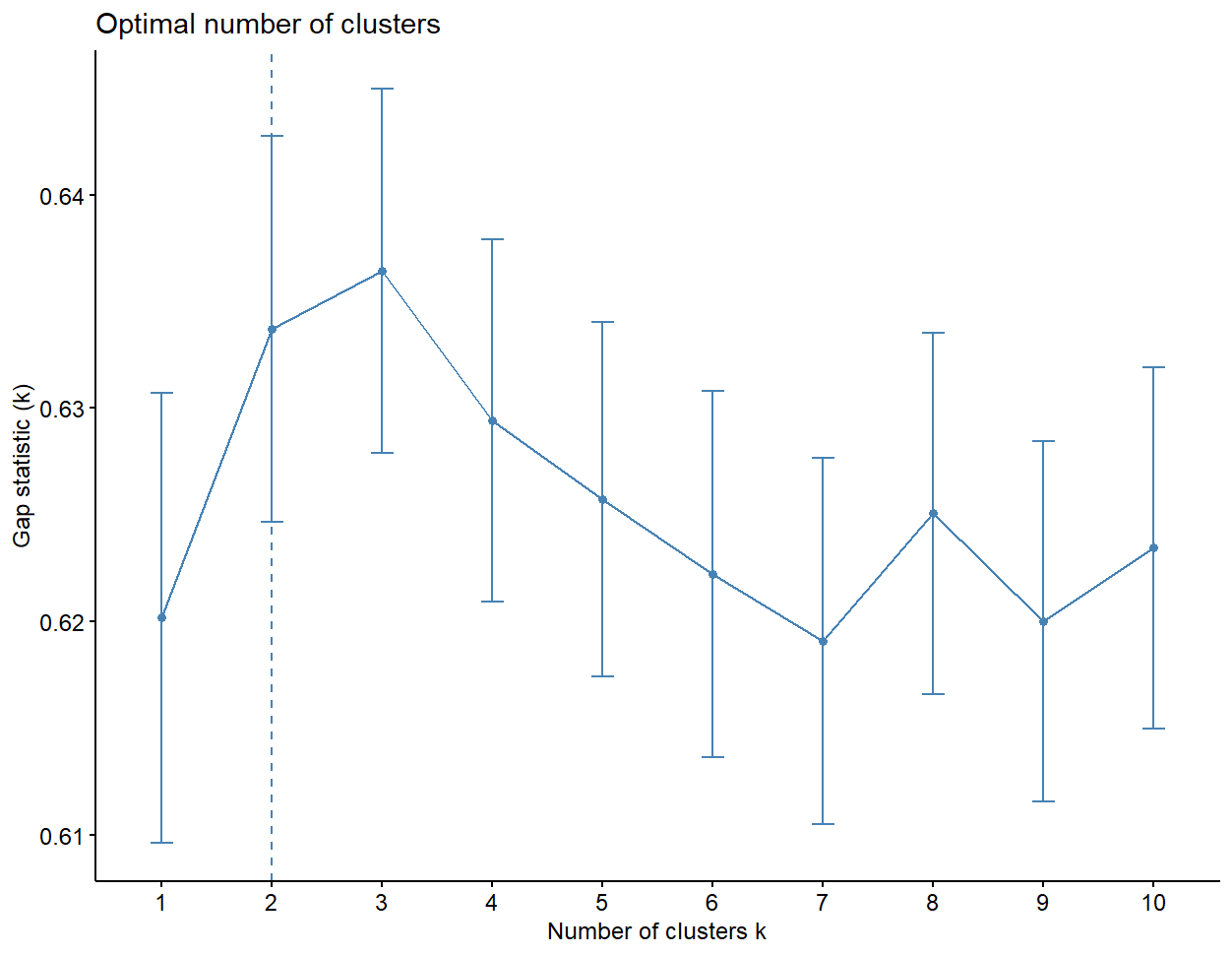

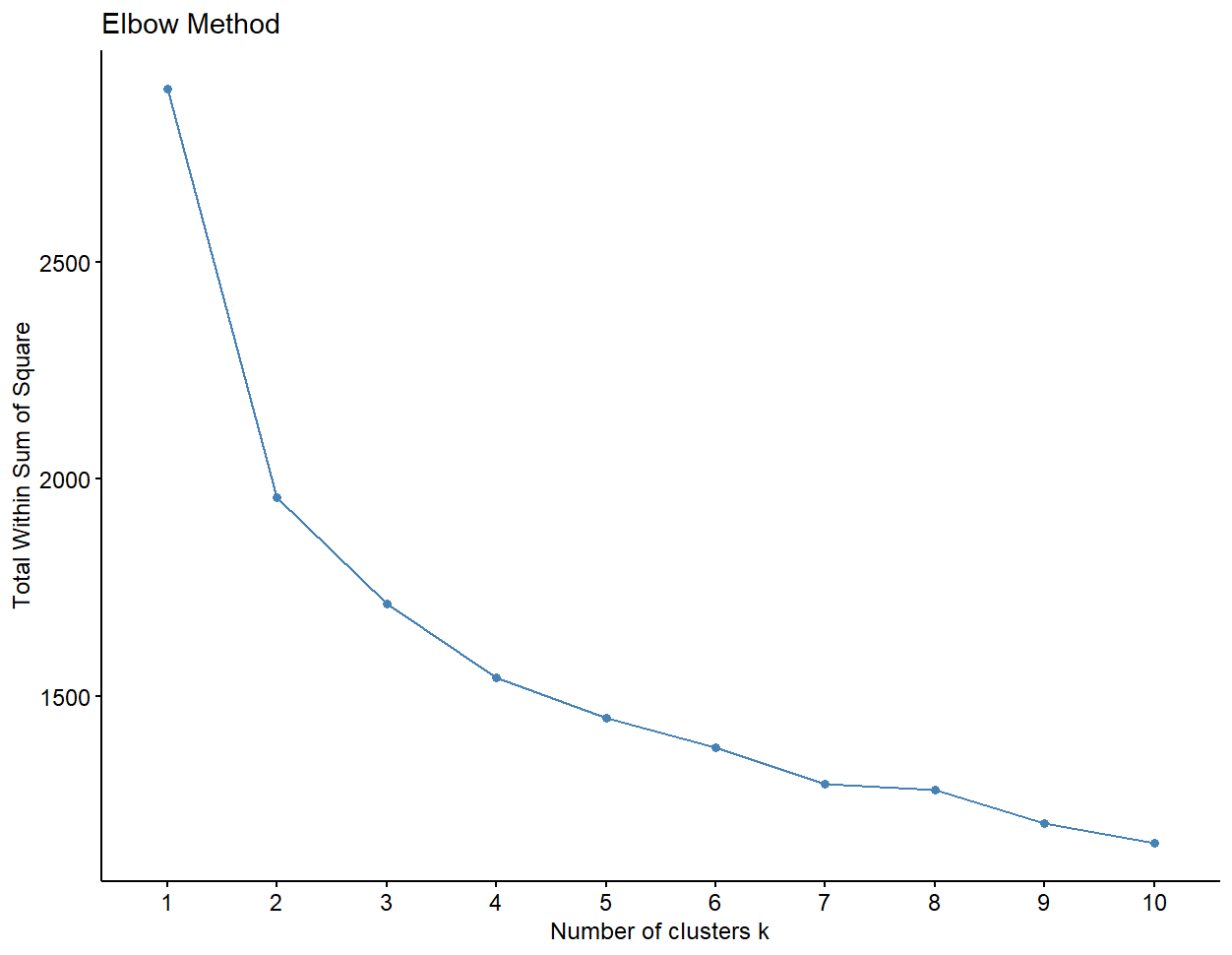
Figure 11: Elbow Method Figure 12: Gap Statistic*

The third step involved the execution of the non-hierarchical K-means algorithm. Using Euclidean distance as the proximity metric, the algorithm iteratively grouped observations by minimizing within-cluster variance, assigning respondents (Cluster 1 = 131; Cluster 2 = 192) based on the similarity of their profiles across the latent constructs.

Cluster profiles were created using the standardized centroid scores across the nine factors (Table 7). This taxonomy was subsequently complemented by an analysis of socio-demographic variables to enrich the characterization of each group. The resulting profiles are presented and discussed in the Results section of the main manuscript.

*Table 7: Clusters composition*

| Cluster | PSGBI_F1 | PSGBI_F2 | PSGBI_F3 | PSGBI_F4 | WRQoL_F1 | WRQoL_F2 | WRQoL_F3 | JETKP_F1 | WDC_F1 |
| --- | --- | --- | --- | --- | --- | --- | --- | --- | --- |
| Cluster 1 | 0,69 | -0,77 | -0,58 | -0,80 | -0,75 | -0,83 | -0,61 | 0,15 | -0,76 |
| Cluster 2 | -0,47 | 0,53 | 0,39 | 0,55 | 0,51 | 0,57 | 0,42 | -0,10 | 0,52 |

Finally, the resulting solution underwent a rigorous validation process. Although the Average Silhouette Width (0.28) reflects a partial overlap in the border regions between groups, the discriminant validity of the partition was confirmed by analyses of variance (ANOVA) (Table 7).

*Table 8: ANOVA Test*

| **Factor** | **Sum Sq (Cluster)** | **Sum Sq (Residuals)** | **F** | **p** | **η2** |
| --- | --- | --- | --- | --- | --- |
| *PSGBI_F1* | 99.41 | 208.56 | 153.00 | <.001 | 0.32 |
| *PSGBI_F2* | 117.76 | 172.50 | 219.10 | <.001 | 0.41 |
| *PSGBI_F3* | 61.38 | 208.51 | 94.50 | <.001 | 0.23 |
| *PSGBI_F4* | 114.11 | 144.80 | 252.90 | <.001 | 0.44 |
| *WRQoL_F1* | 97.90 | 157.30 | 199.80 | <.001 | 0.38 |
| *WRQoL_F2* | 128.42 | 141.10 | 292.20 | <.001 | 0.48 |
| *WRQoL_F3* | 63.55 | 186.19 | 109.60 | <.001 | 0.25 |
| *JETKP_F1* | 4.25 | 271.69 | 5.017 | .026 | 0.02 |
| *WDC_F1* | 107.16 | 167.20 | 205.70 | <.001 | 0.39 |

An examination of the F-statistics and effect sizes (η²) identifies the key variables driving group formation. The WRQoL_F2 factor emerged as the primary driver of the segmentation (F = 292.20; η² = 0.48). Approximately 48% of the variance in this dimension is explained by cluster membership, indicating that the perception of organizational well-being is the most divisive factor within the sample. Furthermore, factors related to perceived gender discrimination, particularly PSGBI_F4 (F = 252.90; η² = 0.44), exhibit high discriminatory power, contributing to the definition of two markedly specular profiles.

The JETKP_F1 (Perspectives for Just Energy Transition Knowledge Production), while remaining statistically significant (p = .026), shows the lowest segregating power (F = 5.01; η² = 0.02). This suggests that while the clusters differ profoundly in terms of well-being and gender bias perceptions, they exhibit a more homogeneous distribution regarding inclusivity policy in the Energy Sector.

In summary, despite an Average Silhouette Width of 0.28, which suggests some overlap between cases at the cluster boundaries, the ANOVA results demonstrate a discriminant validity. The magnitude of the observed effect sizes (with η² reaching 0.48) fully justifies the retention of the two-cluster solution, describing a workforce clearly polarized between a 'positive' and a 'critical' profile.

The density plot (Figure 13) illustrates the distribution of the two clusters across the nine factors. Although a partial overlap can be observed in the central regions of the distribution (consistent with the silhouette width of 0.28), the density peaks, which represent the cluster centroids, remain clearly separated and polarised. This segregation confirms the statistical significance found in the ANOVA tests, with Cluster 1 systematically oriented towards lower organisational well-being profiles than Cluster 2.

*Figure 13: Cluster Density Plot*


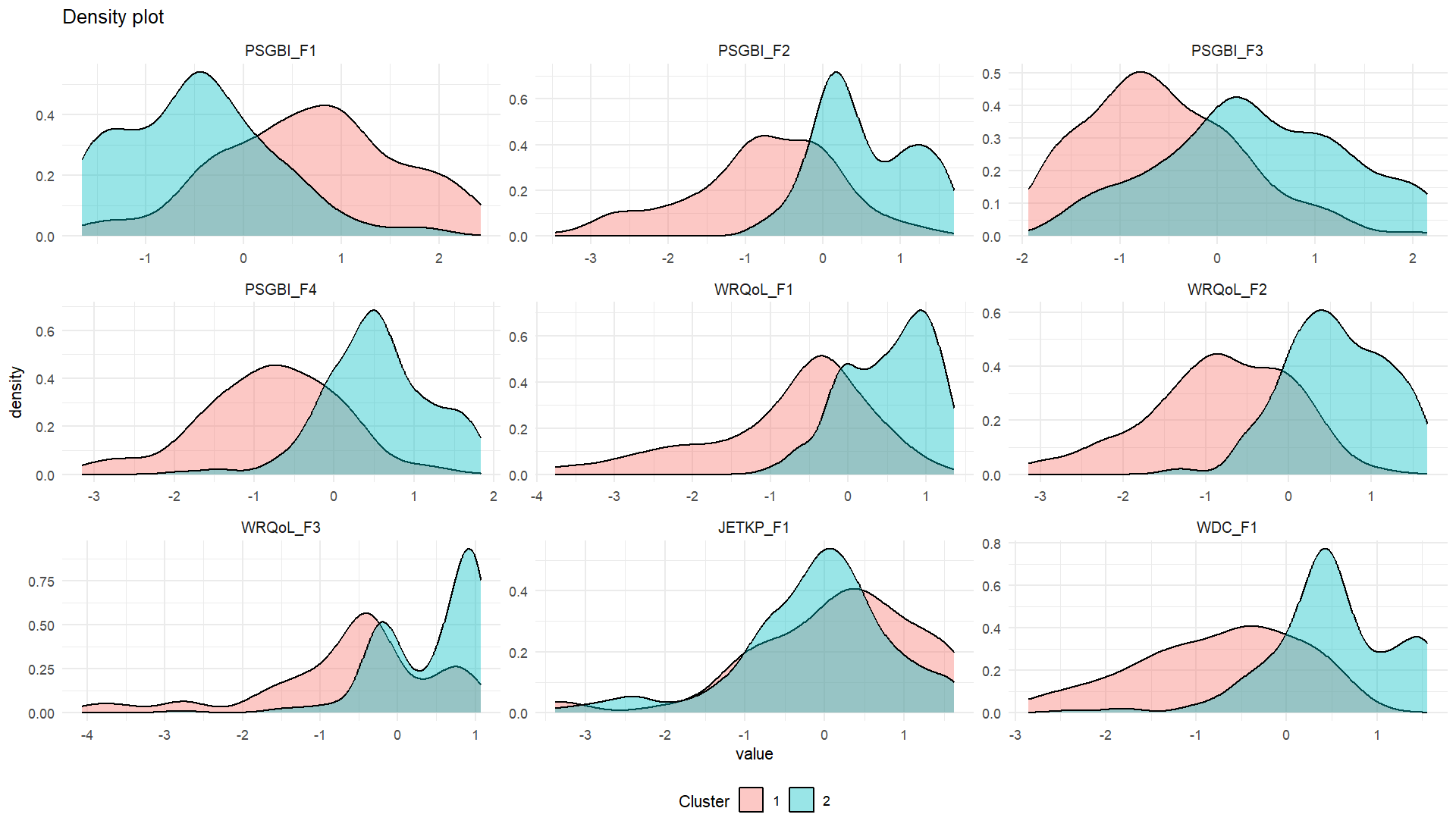


**
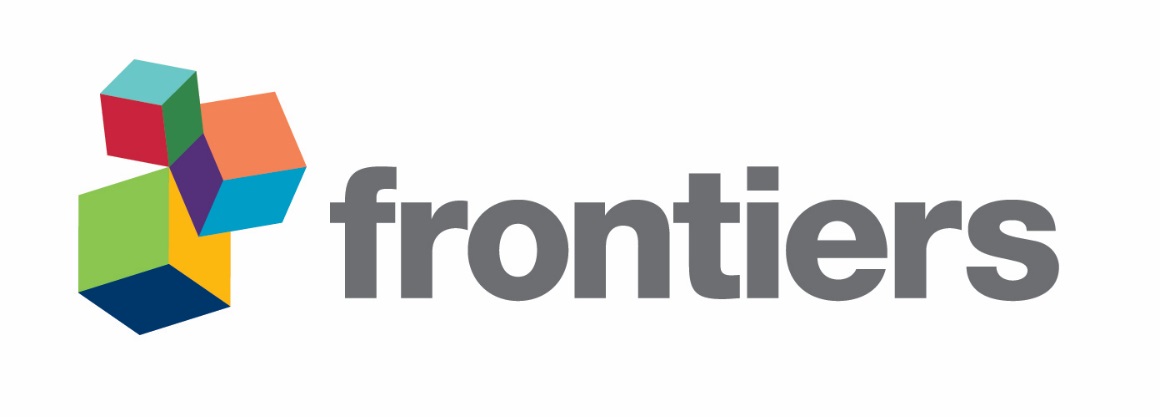
**

1. Easton, S., & Van Laar, D. (2018). User manual for the Work-Related Quality of Life (WRQoL) Scale: a measure of quality of working life. University of Portsmouth. [↑](#footnote-ref-1)
2. Tran, N., Hayes, R. B., Ho, I. K., Crawford, S. L., Chen, J., Ockene, J. K., ... & Pbert, L. (2019). Perceived Subtle Gender Bias Index: Development and validation for use in academia. Psychology of Women Quarterly, 43(4), 509-525. [↑](#footnote-ref-2)
3. Ward, A. K., Beal, D. J., Zyphur, M. J., Zhang, H., & Bobko, P. (2022). Diversity climate, trust, and turnover intentions: A multilevel dynamic system. Journal of Applied Psychology, 107(4), 628. [↑](#footnote-ref-3)
4. Suhr, D. D. (2006). "Exploratory or confirmatory factor analysis?" Proceedings of the Thirty-first Annual SAS Users Group International Conference. [↑](#footnote-ref-4)
